# Immobilization of Recombinant Fluorescent Biosensors Permits Imaging of Extracellular Ion Signals

**DOI:** 10.1021/acssensors.1c01369

**Published:** 2021-11-09

**Authors:** Sandra Burgstaller, Helmut Bischof, Thomas Rauter, Tony Schmidt, Rainer Schindl, Silke Patz, Bernhard Groschup, Severin Filser, Lucas van den Boom, Philipp Sasse, Robert Lukowski, Nikolaus Plesnila, Wolfgang F. Graier, Roland Malli

**Affiliations:** † Gottfried Schatz Research Center, Molecular Biology and Biochemistry, Medical University of Graz, Neue Stiftingtalstraße 6/6, Graz 8010, Austria; ‡ Department of Pharmacology, Toxicology and Clinical Pharmacy, Institute of Pharmacy, Eberhard Karls University of Tuebingen, Auf der Morgenstelle 8, Tuebingen 72076, Germany; § NMI Natural and Medical Sciences Institute at the University of Tuebingen, Reutlingen 72770, Germany; ∥ Gottfried Schatz Research Center, Biophysics, Medical University of Graz, Neue Stiftingtalstraße 6/6, Graz 8010, Austria; ⊥ Department of Neurosurgery, Medical University of Graz, Auenbruggerplatz 29, Graz 8036, Austria; # Laboratory of Experimental Stroke Research, Institute for Stroke and Dementia Research, University of Munich Medical Center, Munich 81377, Germany; ∇ Institute of Physiology I, Medical Faculty, University of Bonn, Bonn 53127, Germany; ○ Munich Cluster for Systems Neurology (SyNergy), Feodor-Lynen-Str. 17, Munich 81377, Germany; ◆ BioTechMed Graz, Mozartgasse 12/II, Graz 8010, Austria

**Keywords:** extracellular ion measurements, recombinant fluorescent biosensors, cell surface immobilization, biotin-traptavidin, FRET, pH-Lemon, GEPII 1.0

## Abstract

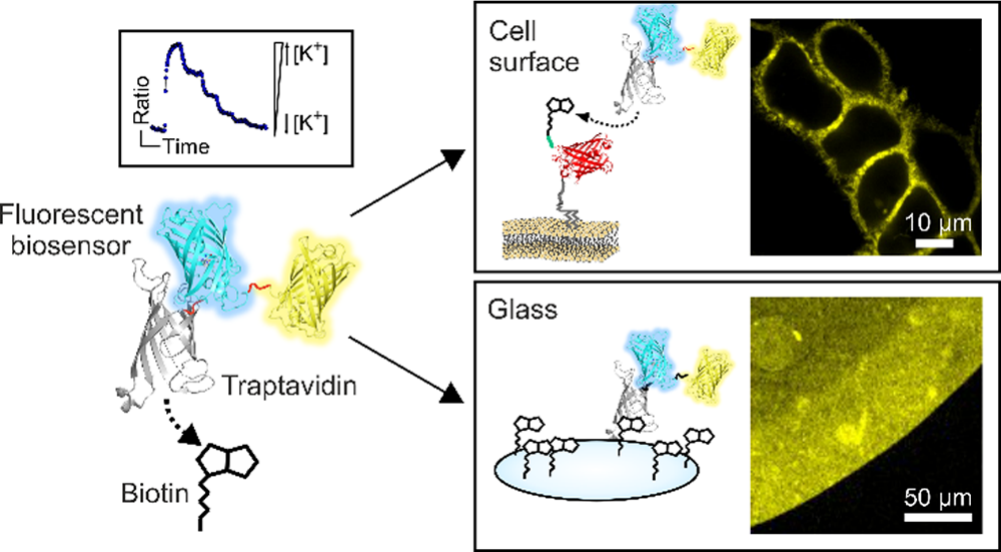

Given the importance of ion gradients and fluxes in biology, monitoring ions locally at the exterior of the plasma membrane of intact cells in a noninvasive manner is highly desirable but challenging. Classical targeting of genetically encoded biosensors at the exterior of cell surfaces would be a suitable approach; however, it often leads to intracellular accumulation of the tools in vesicular structures and adverse modifications, possibly impairing sensor functionality. To tackle these issues, we generated recombinant fluorescent ion biosensors fused to traptavidin (TAv) specifically coupled to a biotinylated AviTag expressed on the outer cell surface of cells. We show that purified chimeras of TAv and pH-Lemon or GEPII 1.0, Förster resonance energy transfer-based pH and K^+^ biosensors, can be immobilized directly and specifically on biotinylated surfaces including glass platelets and intact cells, thereby remaining fully functional for imaging of ion dynamics. The immobilization of recombinant TAv–GEPII 1.0 on the extracellular cell surface of primary cortical rat neurons allowed imaging of excitotoxic glutamate-induced K^+^ efflux in vitro. We also performed micropatterning of purified TAv biosensors using a microperfusion system to generate spatially separated TAv–pH-Lemon and TAv–GEPII 1.0 spots for simultaneous pH and K^+^ measurements on cell surfaces. Our results suggest that the approach can be greatly expanded by immobilizing various biosensors on extracellular surfaces to quantitatively visualize microenvironmental transport and signaling processes in different cell culture models and other experimental settings.

An elegant approach to monitor intra- and extracellular signaling events is to use genetically encoded fluorescent biosensors that can be well targeted to cell organelles.^1,2^ Targeting fluorescent protein (FP)-based biosensors to the outer leaflet of the plasma membrane is usually achieved by fusing them with N-terminal secretion signals and C-terminal plasma membrane anchoring domains.^2,3^ Such targeting strategies have been used to successfully target biosensors for small-molecule (neuro)transmitters including γ-aminobutyric acid,^4,5^ glutamate,^6^ ATP,^2^ or serotonin^7^ to the cell surface. In a previous study, we used such a strategy to target pH-Lemon, a pH reporter, to the exterior of the plasma membrane using an N-terminal secretory leading sequence and a glycosylphosphatidyl (GPI)-anchored moiety.^3,8,9^ However, such targeting signals induce the entering of biosensors into the endoplasmatic reticulum (ER), further sorting in the Golgi apparatus and traveling to the cell surface within secretory vesicles. Endocytosis can then bring the plasma membrane-targeted biosensor back into intracellular vesicular structures. Thus, the use of secretion signals and plasma membrane anchoring domains results in strong labeling of intracellular vesicles,^3^ which can complicate the selective investigation of extracellular activities on cell surfaces. Here, we tested cell surface-targeted pH-Lemon and GEPII 1.0, a Förster resonance energy transfer (FRET)-based pH and K^+^ biosensor,^3,10^ respectively, which we have developed recently. As expected, both GPI-anchored biosensors were also found predominately in intracellular structures.^3,8^ Moreover, while pH-Lemon–GPI remained pH-sensitive, we here found that the GPI-anchored K^+^ biosensor completely lost its functionality. Thus, we devised an alternative strategy for the application of both biosensors on the outer leaflet of the plasma membrane for extracellular cell surface pH and K^+^ imaging, which might be important and interesting in cancer research^11,12^ and when using excitable cells such as neurons.^13^

A quite distinct strategy to bring biosensors to the extracellular microenvironment is to immobilize the purified, fluorescent tools on the cell surface, thereby omitting sensors traveling through the secretory pathway. This might allow the usage of biosensors on cell surfaces without unwanted intracellular accumulations and modifications. Such an approach has indeed already been used successfully to immobilize biotinylated fluorescent amino acid biosensors on biotinylated cell surfaces via streptavidin, creating a biotin–streptavidin–biotin sandwich.^14−16^ Here, we worked out another simplified biotinylation-based technique to directly, selectively, and functionally anchor recombinant biosensor constructs on biotinylated surfaces.^8^ Instead of fusing a biotin tag to the biosensor, we directly fused it with traptavidin (TAv),^17^ an improved streptavidin variant. We here show that the newly engineered recombinant biosensors for pH^3^ and K^+^^10^ fused with TAv could be directly coupled to biotinylated glass surfaces and remain functional.^8^ The biosensor constructs could also be selectively and directly immobilized on cells of interest that expressed a cell surface-targeted biotinylated AviTag.^8,18,19^ Our study suggests that this strategy can easily be expanded to other biosensors in various applications.

## Experimental Methods

### Substances and Buffers

All substances and buffers are mentioned in the Supporting Information.

### Plasmids, Targeting and Sequences

Plasmids, targeting strategies and sequences are mentioned and explained in the Supporting Information.

### Characterization of Recombinant Proteins

A detailed description of the protocol can be found in the Supporting Information.

### Fluorescence Microscopy

Table S3 gives an overview of microscopes used for the different measurements.

### Cell Culture and Transfection

Cell culture and transfection methods are explained in the Supporting Information.

### Immobilization of TAv–pH-Lemon and TAv–GEPII 1.0

The biotinylated glass slides (Microsurfaces Inc., New Jersey, USA) were incubated with the sensor solution for 20 min and washed with phosphate-buffered saline (PBS) after incubation. Alternatively, a 2 μL drop containing the sensor solution was added to the glass slide and air-dried for approximately 15–20 min. The glass slide was washed with PBS to remove unbound sensors. Cells were washed thrice with PBS. For coupling the whole well, 500 μL of the protein solution (8–10 μM final concentration) was added to the cells and incubated at 25 °C for 20 min in the dark. The cells were washed thrice with PBS and kept in physiological buffer for imaging. For micropatterning, a BioPen^20^ (Fluicell, Mölndal, Sweden) was used for microperfusion. The cells were placed on the microscope and covered with 1.5 mL of physiological buffer. The BioPen tip was loaded with the TAv sensor protein solutions and positioned near the cells of interest using a micromanipulator (Sutter MP-225; Sutter Instruments, Novato, CA, USA). The cells of interest were perfused for up to 7 min with either TAv–pH-Lemon or TAv–GEPII 1.0. The tip was removed from the well after immobilization.

### Data Processing and Statistical Analysis

A detailed description of the analysis and statistics is provided in the Supporting Information.

## Results and Discussion

### GPI-Anchored Biosensors Accumulate in Intracellular Structures and Can Lose Functionality

**Figure 1:**
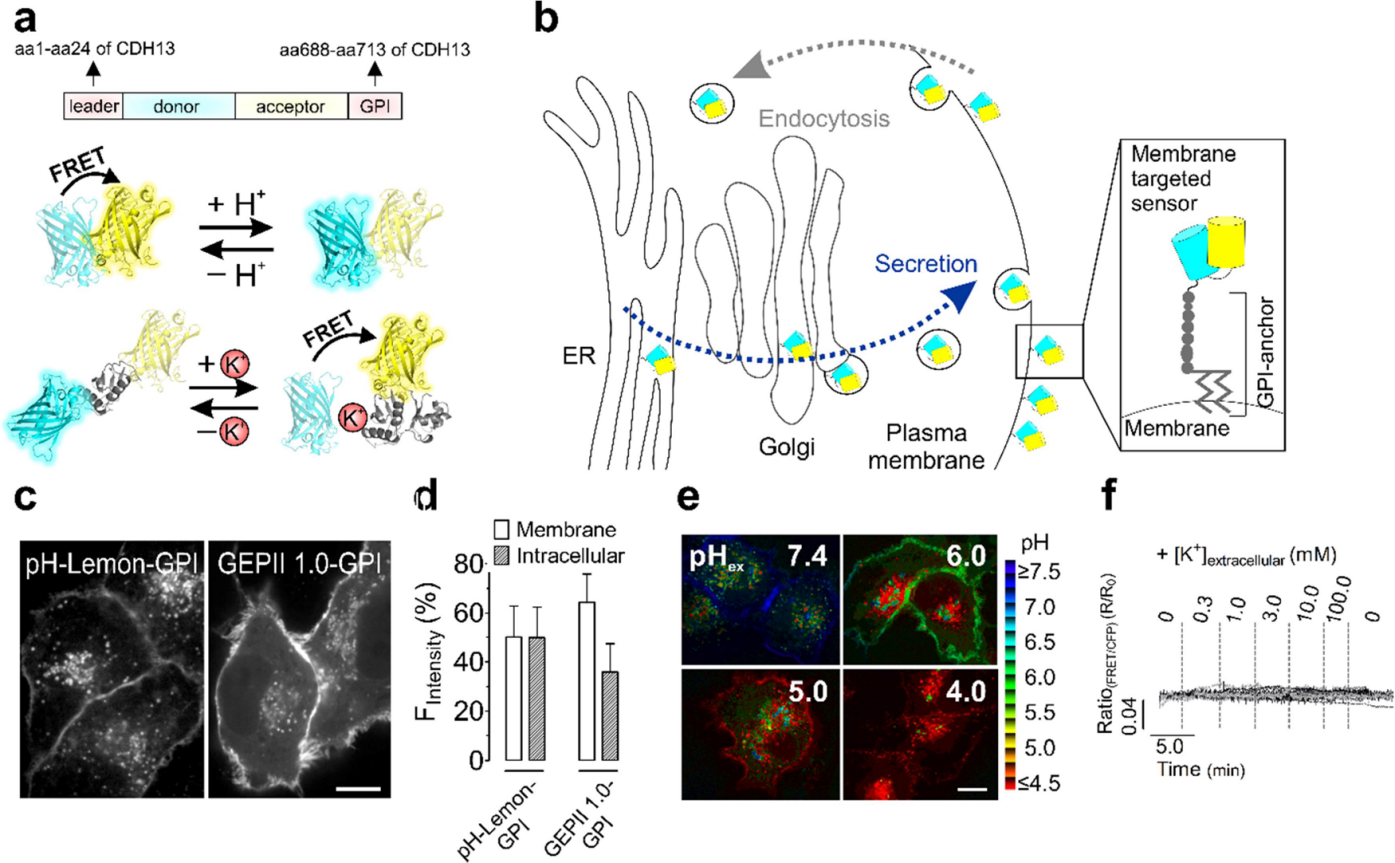
GPI anchoring of FRET-based biosensors for targeting to the outer leaflet of the plasma membrane leads to intracellular sensor accumulation and loss of function. (a) Schematic drawing of plasma membrane targeting of FRET-based biosensors using an N-terminal leader sequence (i.e., the 24 amino acids of the CDH13 targeting sequence) and a C-terminal GPI-anchor attachment signal (i.e., the 26 amino acids of GPI-anchor domain of CDH13) and (lower panels) schematic illustration of pH-Lemon and GEPII 1.0, a pH and K^+^ biosensor, respectively, with different working principles. (b) Schematic drawing of a genetically encoded FRET-based plasma membrane targeted biosensor traveling through the ER, the Golgi, and secretory vesicles upon GPI anchoring. Eventually, sensors are internalized via endocytosis. (c) Representative ACLSM images (mTurquoise2 and mseCFP fluorescence) of HeLa cells transiently expressing pH-Lemon-GPI or GEPII 1.0-GPI. Scale bar represents 10 μm. (d) Quantitative analysis of ACLSM images (one *z* plane) to assess the abundance of the biosensors at the plasma membrane or within intracellular structures. Columns represent the average cellular fluorescence distribution ± SD of pH-Lemon-GPI and GEPII 1.0-GPI on the plasma membrane and intracellular structures (*n* = 16 cells for pH-Lemon-GPI, and *n* = 15 cells for GEPII 1.0-GPI). (e) Pseudo-colored FRET ratio ACLSM images of HeLa cells expressing pH-Lemon-GPI in the presence of extracellular buffers with different pH values as indicated in the images. Scale bar represents 10 μm. (f) Normalized FRET ratio signals over time of HeLa cells expressing GEPII 1.0-GPI in response to increasing [K^+^]_ex_ from 0 to 100.0 mM measured using widefield microscopy. Data represent 31 cells of 4 experiments.

We first confirmed strong intracellular accumulations of FRET-based biosensors for pH and K^+^ (Figure 1a) upon targeting them to the cell surface by GPI anchoring (Figure 1a,b), regardless of the transfection method (Figures 1c,d and S1a–c) or the construct size containing only one FP (Figure S1d). The intracellular abundance of biosensors (Figure 1c,d) complicates the selective discrimination of extra- versus intracellular pH alterations since lowering the extracellular pH also affected the pH of intracellular vesicles of cells expressing pH-Lemon–GPI (Figure 1e).^3^ Importantly, although GPI anchoring did not affect the sensor functionality of pH-Lemon^3^ (Figure 1e), GEPII 1.0–GPI completely lost its K^+^ sensitivity (Figure 1f).^8^ We hypothesized that N-glycosylation, a frequent posttranslational modification within the secretory pathway,^21^ of GEPII 1.0–GPI rendered it K^+^-insensitive and confirmed the glycosylation of the construct by western blot (Figure S2a–c).

Also, while cytosolic GEPII 1.0 responded to K^+^ changes as expected (Figure S2d,e), targeting GEPII 1.0 into the ER lumen, the entry gate of the secretory pathway, already impaired K^+^ sensing (Figure S2f,g). Considering that the functionality of pH-Lemon–GPI (Figure 1e) but not of GEPII 1.0–GPI (Figure 1f) was maintained, we hypothesized that glycosylation, possibly within the K^+^ binding domain at N36 and N76 (Figure S2a,b), impaired the functionality of the GPI-anchored K^+^ biosensor.^22^

The vesicular abundance of plasma membrane-targeted biosensors did not appear problematic for other sensors and experimental settings.^2,23^ However, we here aimed to tackle these issues in the case of both biosensors, pH-Lemon and GEPII 1.0, to accomplish pH and K^+^ measurements at the extracellular surface of the plasma membrane of cells of interest. Thus, we came up with an improved biotinylation-based approach to immobilize the recombinant constructs specifically to cell surfaces.

### Recombinant TAv Biosensors Dissolved in Solution and Immobilized on Glass Remain Functional

**Figure 2:**
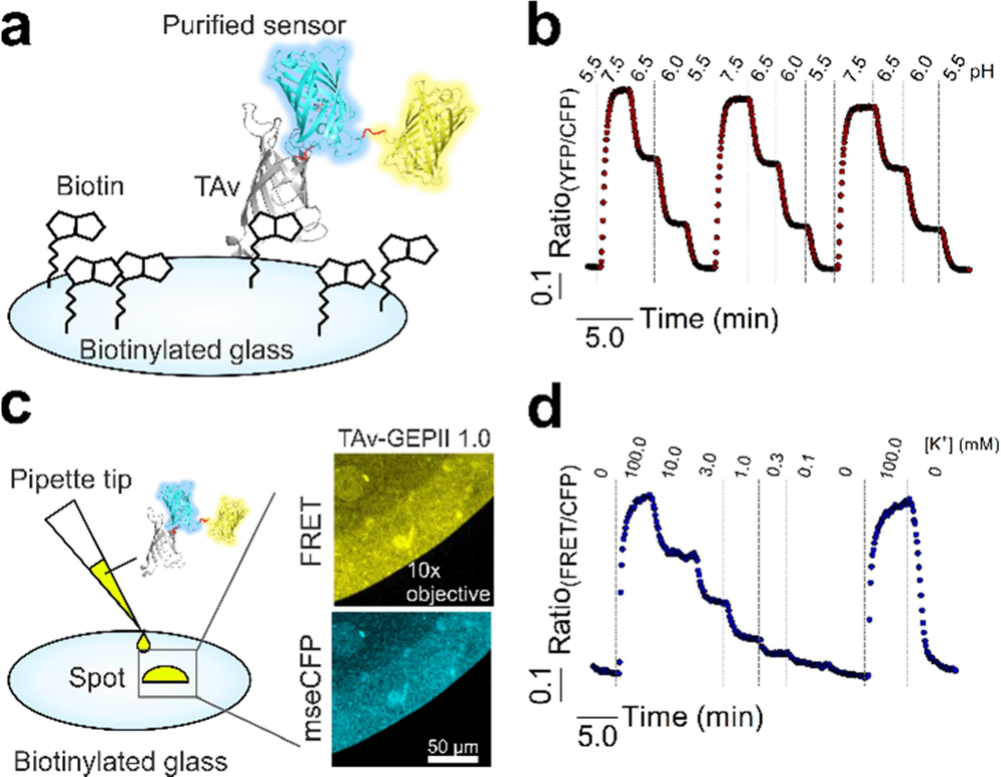
Recombinant FRET-based TAv biosensors for pH and K^+^ remain functional upon immobilization on biotinylated glass slides. (a) Illustration of a coupled biosensor to a biotinylated glass surface. (b) Representative response of glass-coupled TAv–pH-Lemon perfused with buffers with different pH values after lyophilization and storage for 24 h. (c) Schematic drawing and representative widefield images of the application of a 2 μL sensor spot of TAv–GEPII 1.0 immobilized on biotinylated glass slides by a conventional pipette tip. (d) Representative FRET ratio signal of TAv–GEPII 1.0 immobilized on biotinylated glass upon perfusion with different [K^+^] buffers (0–100 mM; *n* = 3).

To omit intracellular sensor traveling but localize the biosensors to the cell surface as recombinant proteins, we designed and purified recombinant biosensors. Recombinant pH-Lemon and GEPII 1.0 were directly fused with TAv, a mutated and improved streptavidin analogue,^17^ in order to directly bind the sensor proteins to biotinylated surfaces (Figure 2a). Before immobilizing them on biotinylated glass or cells, we first examined the functionality and affinity of the recombinant biosensor TAv-fusion constructs dissolved in buffered solution. Recombinant TAv–pH-Lemon (Figure S3a,c) and TAv–GEPII 1.0 (Figure S3b,c) in a solution showed clear FRET ratio changes in response to pH and K^+^ alterations (Figure S3d,e), respectively,^8^ without affecting their sensitivities (Table S4). When immobilized on biotinylated glass for measuring the functionality and stability (Figure 2a,c), both biosensor constructs (Figure 2b,d) showed clear FRET ratio changes in response to respective fluctuations of ion concentrations. Even after lyophilization and storage of glass slides loaded with pH-Lemon, FRET ratio changes to pH alterations could be recorded (Figure 2b), pointing to the high stability and robustness of the recombinant biosensors and the TAv-biotin interaction on glass. These experiments might also pave the way to the development of novel smart devices for diagnosis and research exploiting a variety of immobilized FP-based biosensors.

### Plasma Membrane-Coupled TAv Biosensors Are Functional and Report Glutamate-Induced K^+^ Efflux from Neurons

**Figure 3:**
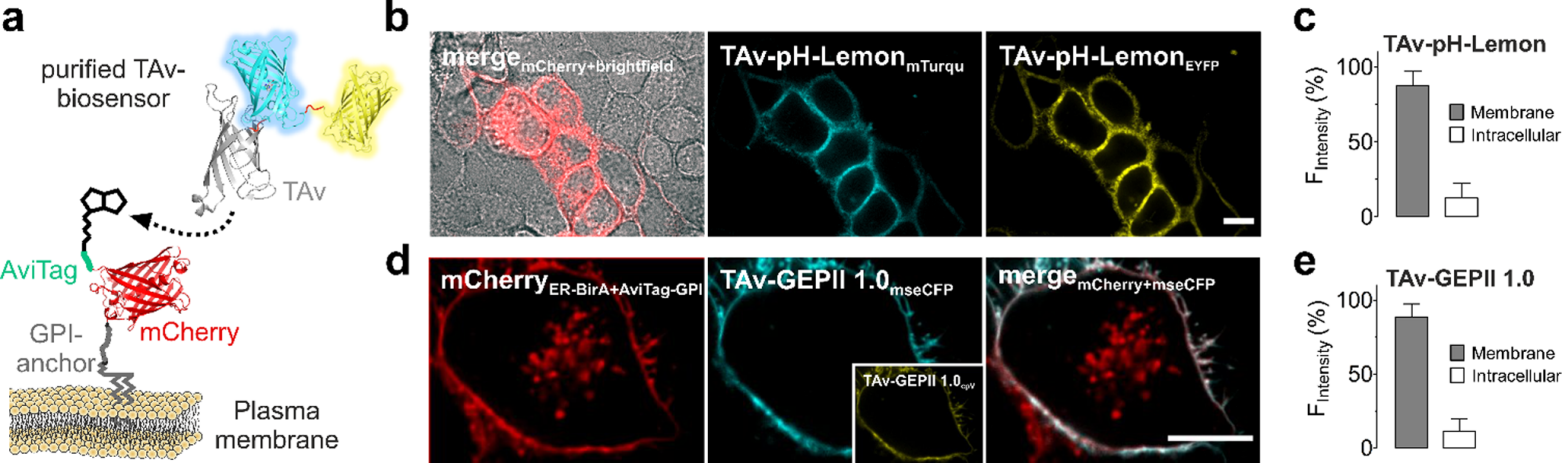
Recombinant FRET-based TAv biosensors specifically couple to biotinylated cell membranes of intact cells. (a) Schematic drawing of TAv-fused biosensors binding to the biotinylated AviTag present at the outer cell membrane. (b) High-resolution ACLSM images (one *z* plane) of HeLa cells expressing AviTag-mCherry-GPI and ER-BirA-mCherry (left image, a merge of a brightfield image and cells expressing AviTag-mCherry-GPI and ER-BirA-mCherry) coupled with TAv–pH-Lemon (middle image, TAV-pH-Lemon-mTurquoise2 and right image, TAV-pH-Lemon-EYFP). The scale bar represents 10 μm. (c) Analysis of one *z* plane of ACLSM images to quantify the cellular fluorescence distribution of pH-Lemon–GPI between the plasma membrane and intracellular structures. Columns represent average fluorescents intensities ± SD at the plasma membrane and of intracellular structures; *n* = 41 cells. (d) High-resolution ACLSM images (one *z* plane) of HeLa cells expressing AviTag-mCherry-GPI and ER-BirA-mCherry (left image) loaded with TAv–GEPII 1.0 (big middle image and small middle image). The right image displays a merge of AviTag-mCherry-GPI/ER-BirA-mCherry and TAv–GEPII 1.0-mseCFP. The scale bar represents 10 μm. (e) Quantitative analysis of one *z* plane of ACLSM images of fluorescence of GEPII 1.0-GPI in HeLa cells. Columns show the average fluorescence distribution of GEPII 1.0-GPI at the plasma membrane and within intracellular structures (mean ± SD, *n* = 18 cells).

We next aimed to immobilize the TAv biosensors on the surface of living cells (Figure 3a). To selectively express a biotinylated tag on cell surfaces of cells of interest, we modified an established approach^18,19^ and generated two separate mCherry-labeled constructs. One consisted of the plasma membrane-targeted AviTag^24^ (AviTag-mCherry-GPI) (Figure S4a,c,e), and the other consisted of ER-BirA-mCherry, the ER-targeted biotin ligase^18^ (Figure S4b,d,e). The co-expression of the two constructs should result in a biotinylation of the AviTag by BirA when the AviTag traversed the ER lumen upon membrane targeting (Figure S4c−e).

Expectedly, the biotinylated AviTag efficiently coupled with the purified recombinant TAv–pH-Lemon (Figure 3b) and TAv–GEPII 1.0 (Figure 3d) with minimal intracellular background fluorescence under these conditions (Figure 3c,e). Notably, we here used cells for less than 1 h at room temperature and directly after coupling the biosensors to the biotinylated cell surface. However, the internalization of cell surface-coupled biosensors by endocytosis at higher temperatures in in vitro and in vivo application over time is expectable.^25,26^ It would be interesting to test if internalized TAv–GEPII 1.0 remains functional over time. Importantly, no sensor fluorescence signal could be detected on the cell surface of mCherry–negative cells, indicating the specificity of the coupling process (Figure 3b).^8^

**Figure 4:**
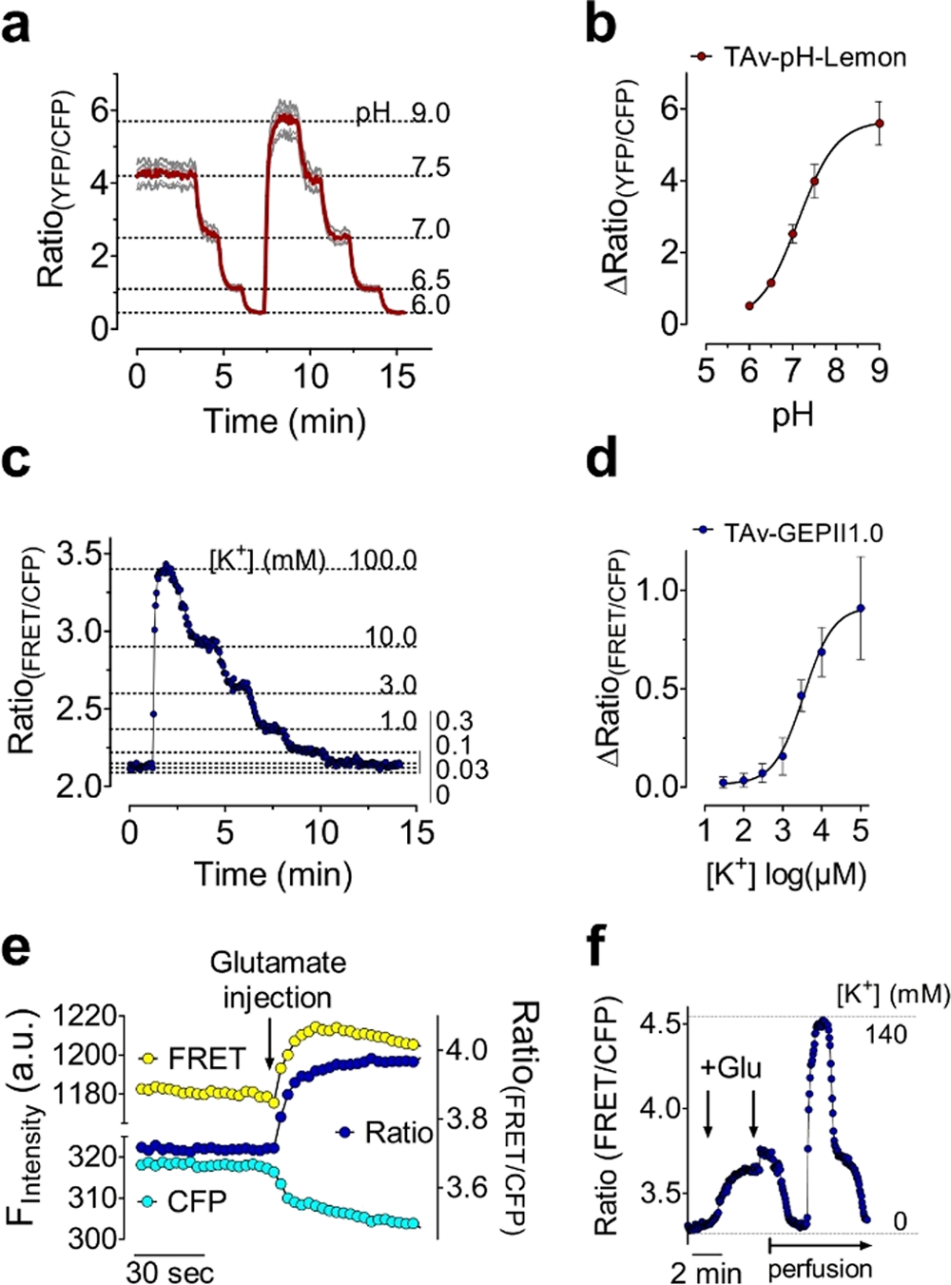
Recombinant FRET-based TAv biosensors coupled to biotinylated cell surfaces are functional and report ion alterations in situ. (a) Average curve (red) and single cell responses (gray curves) of FRET ratio signals of TAv–pH-Lemon immobilized on HeLa cells that expressed AviTag-mCherry-GPI and ER-BirA-mCherry. Signals were measured using widefield microscopy. Buffers of different pH values were perfused as indicated (*n* = 3 independent experiments). (b) Concentration–response curve of TAv–pH-Lemon coupled to the surface of HeLa cells. The data are expressed as a mean ± SD (*n* = 3 and 18 cells). (c) Representative FRET ratio signal of TAv–GEPII 1.0 over time. The recombinant biosensor was coupled to a HeLa cell expressing AviTag-mCherry-GPI and ER-BirA-mCherry. The fluorescence was quantified over time using widefield microscopy. [K^+^]_ex_ was increased or decreased as indicated using gravity-based perfusion (*n* = 4). (d) Concentration–response curve of TAv–GEPII 1.0 coupled to the surface of HeLa cells. The data are expressed as a mean ± SD (*n* = 4 and 15 cells). (e) FRET, cyan fluorescent protein (CFP), and respective ratio signals over time of TAv–GEPII 1.0 immobilized on primary rat neurons upon addition of glutamate. (f) Response of TAv–GEPII 1.0 immobilized on the surface of primary rat neurons in response to glutamate injections. First, the perfusion was stopped, and two glutamate boli were injected into 0 mM K^+^ buffer to reach a final concentration of 500 μM and 1 mM as indicated (arrows). Subsequently, the sensor functionality was checked by starting perfusion to transiently elevate extracellular K^+^ from 0 to 140 mM K^+^.

To test the functionality of TAv–pH-Lemon (Figures 4a,b and S5a,b) and TAv–GEPII 1.0 (Figures 4c,d and S5c,d) the TAv biosensors were immobilized on the cell surface of HeLa cells that expressed the biotinylated AviTag. Cells were then perfused with experimental buffers with pH levels ranging from 6.0 to 9.0 (Figures 4a,b and S5b) or different [K^+^] (Figures 4c,d and S5d). Upon perfusion, the TAv sensors responded to pH and K^+^ alterations in a fast, ratiometric, and reversible manner (Figures 4a–d and S5b,d). The same TAv biosensor fluorescence was found on the cell periphery after 10 min of rinsing the cell with buffer using a perfusion system (Figure S5e), pointing again to the stable interaction between the recombinant sensor and the biotinylated cell surface tag. Although K^+^ sensing of GEPII 1.0 relies on a conformational rearrangement of the K^+^ sensing domain^10^ (Figure 1a), immobilization did not hamper the K^+^ measurements (Figure 4c,d). However, the sensitivity (EC_50_) and dynamic range of the TAv biosensors were significantly reduced upon immobilization (Table S4). The immobilization of TAv–GEPII 1.0 on cells also increased basal FRET ratio values even in the absence of extracellular K^+^ in the buffer (Table S4). This observation might point to K^+^-insensitive intermolecular FP interactions due to the spatially close sensor coupling. The mean pKa of coupled TAv–pH-Lemon on cells was 7.12 (range: pH 7.03–pH 7.20) (Figure 4b and Table S4), and the mean EC_50_ of cell surface-coupled TAv–GEPII 1.0 was 3.46 mM (range: 2.59–4.62 mM) (Figure 4d and Table S4). According to previous studies, the pKa values of TAv–pH-Lemon and the EC_50_ of TAv–GEPII 1.0 seem to be well suitable for the detection of extracellular pH and K^+^ alterations.^27,28^

Because K^+^ plays a major role in neuronal homeostasis,^29^ we next tested our approach for monitoring K^+^ efflux from primary neurons in response to glutamate. Considering the effect of high glutamate levels being accompanied by strong K^+^ efflux,^30^ we examined whether this glutamate-induced K^+^ efflux can be optically detected using TAv–GEPII 1.0 coupled to the cell surface of primary neurons (Figure 4e,f) expressing ER-BirA and AviTag-mCherry-GPI (Figure S6a). We, therefore, used a high-pressure microperfusion system^20^ to load selected mCherry-positive cells with purified TAv–GEPII 1.0 directly on the fluorescence microscope (Figures S6b,c and S7a). To prevent fast dilution of extracellular K^+^, we stopped the perfusion and added a cytotoxic glutamate bolus to the neurons by injection (Figure 4e,f). The first injection of glutamate instantly resulted in a rapid increase in the FRET ratio signal of the cell surface-coupled K^+^ biosensor, indicating massive K^+^ efflux from neurons in response to the excitatory neurotransmitter (Figures 4e,f and S7b). A second injection only slightly further increased the FRET ratio signal, indicating that the primary neurons were already strongly stimulated (Figure 4f). As expected, a subsequent start of the perfusion reversed the effect (Figure 4f). Subsequent K^+^ addition and removal rapidly and strongly changed the FRET ratio signal of the cell surface-coupled K^+^ biosensor, confirming its functionality (Figure 4f).

### Micropatterning of TAv Biosensors Allows for Simultaneous Imaging of pH and K^+^ in the Same Experimental Setting

**Figure 5:**
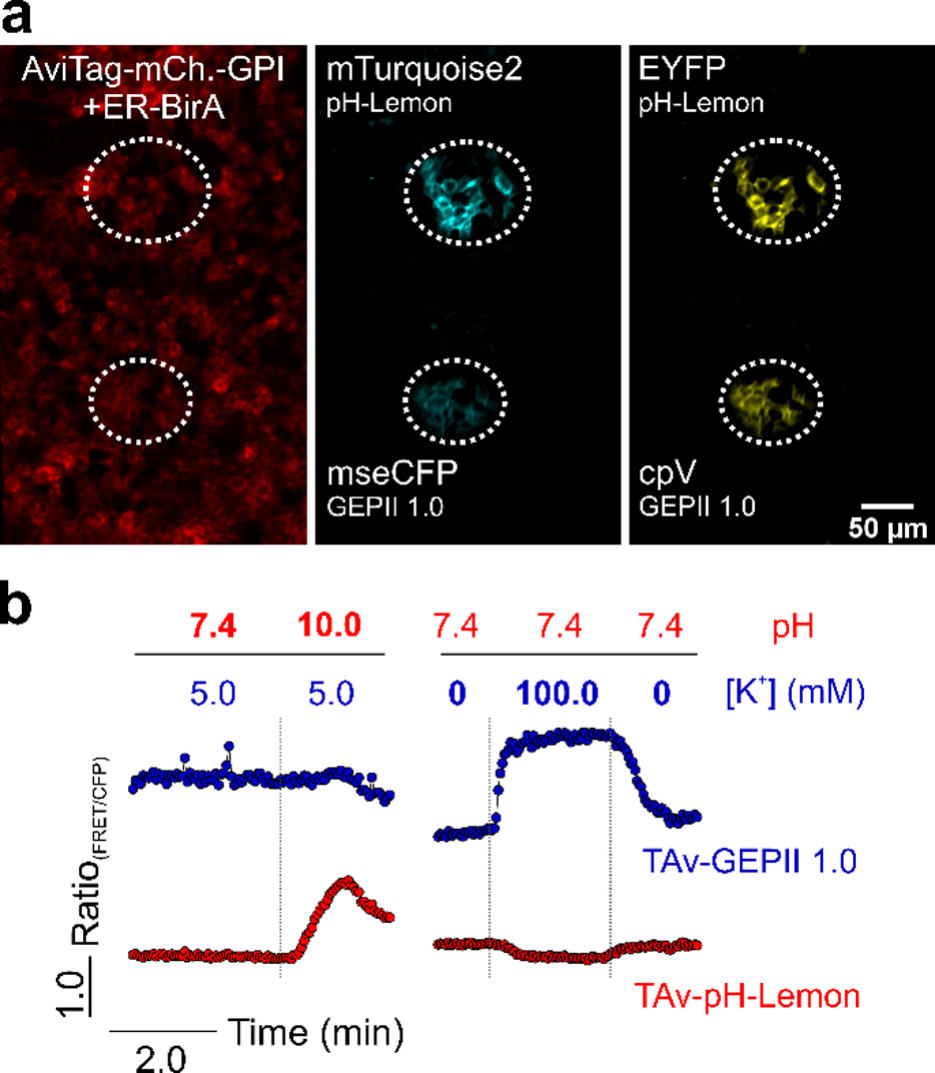
Micropatterning of recombinant FRET-based TAv biosensors allows for co-imaging of pH and K^+^ in the same experimental setting. (a) Representative widefield fluorescence images of INS-1832/13 cells expressing AviTag-mCherry-GPI and ER-BirA (left). Two distinct, separated sensor spots of purified TAv–pH-Lemon and TAv–GEPII 1.0, respectively, were generated using microperfusion (white dashed circles), both positive for cyan (middle) and yellow fluorescent protein (right). (b) FRET ratio signal of surface-coupled TAv–pH-Lemon and TAv–GEPII 1.0 over time. The cells carrying the recombinant sensors were first perfused with buffers with different pH levels (red) and a constant K^+^ concentration (blue) and subsequently with different K^+^ concentrations and the same pH level as indicated.

To co-image pH and K^+^ with the spectrally identical biosensors in one experimental setting, we next used a micropatterning approach. The usage of the high-pressure microperfusion^20^ device allowed us to “paint” spatially separated microspots of TAv–pH-Lemon and TAv–GEPII 1.0 on cells expressing the biotinylated GPI-anchored AviTag. (Figures 5a and S6b,d,e). FRET ratio signals from both spots containing the two different FRET-based biosensors were recorded simultaneously over time to test the suitability for co-imaging of pH and K^+^ alterations (Figure 5b). Upon addition of buffers with different [K^+^] (but the same pH), TAv–GEPII showed fast ratiometric changes, whereas TAv–pH-Lemon was only minimally affected (Figure 5b). Vice versa, with the addition of buffers with different pH values (but the same [K^+^]), TAv–pH-Lemon responded with clear FRET ratio changes, while TAv–GEPII was only little affected (Figure 5b). Such multiparametric imaging of local cell surface pH and K^+^ alterations exploiting the purified TAv biosensors in tumors in vivo might become possible in future. Such an approach might be also used to co-detect and correct for pH fluctuations, which have the potency to perturb the performance of most FP-based biosensors.^31^

## Conclusions and Outlook

While purified biosensors have mainly been used for sensor characterization purposes,^3,10,32,33^ we describe a novel biotinylation-based procedure to use genetically encoded sensors for the recording of extracellular ions locally on cell surfaces and glass slides upon their immobilization using three workflows (Figure S8): first, purification of the recombinant TAv biosensor, second, expression of the biotinylated cell surface tag or using a biotinylated glass slide, and third, coupling of the TAv biosensor to the biotinylated surface.

Considering the remaining functionality and precise targeting of recombinant TAv biosensor constructs with different working principles (conformational rearrangement vs intrinsic sensitivity), this technique might be expanded to immobilize a variety of different ratiometric and intensiometric biosensors. The precise application using microperfusion might furthermore allow for the immobilization of sensor protein on selected cells (types) (e.g., on tumor slices). While extracellular ion alterations might be hard to capture in a big extracellular volume in vitro, this technique might be further used in interesting experimental settings with reduced volume such as in the extracellular area of mouse brains. However, the meaningful usage of the recombinant fluorescent biosensors in vivo might be challenging and requires further research.

## Supporting Information Available

Substances, protein purification buffers, protein purification, buffers for in vitro fluorimetry, cell culture and transfection, buffers for fluorescence microscopy, fluorescence microscopy, western blot analysis, glutamate addition to primary rat neurons, data processing, statistical analysis, list of all plasmids/vectors used for mammalian and bacterial expression, sequences of targeting signals and constructs used, overview of the microscopes used for measuring GEPII and pH-Lemon (fusion) constructs, comparison of targeted and immobilized pH-Lemon and GEPII 1.0 variants, GPI targeting of biosensors leads to sensor accumulation within the secretory pathway, biosensor localization within the secretory pathway impacts sensor functionality, possibly due to glycosylation, characterization of TAv-fused sensors in solution, constructs and scheme for the immobilization of TAv-fused biosensors, functionality of TAv-pH-Lemon and TAv–GEPII 1.0 after immobilization, local immobilization of TAv sensors using microperfusion, visualization of glutamate-induced K^+^ efflux from primary neurons using surface-coupled TAv–GEPII 1.0, and cell and sensor preparation and application to living cells (PDF)
