# Supplementary material for: Immobilization of Recombinant Fluorescent Biosensors Permits Imaging of Extracellular Ion Signals
Source: ACS Sens. 2021 Nov 9;6(11):3994–4000. doi: 10.1021/acssensors.1c01369 (PMC8630794; doi:10.1021/acssensors.1c01369)
Supplement: Supplementary file 1 — se1c01369_si_001.pdf [file se1c01369_si_001.pdf]

Supplementary Information

Immobilization of recombinant fluorescent biosensors permits imaging of extracellular ion signals

Sandra Burgstaller[1,2,3], Helmut Bischof[1,2], Thomas Rauter[1], Tony Schmidt[4], Rainer Schindl[4], Silke Patz[5], Bernhard Groschup[6], Severin Filser[6], Lucas van den Boom[7], Philipp Sasse[7], Robert Lukowski[2], Nikolaus Plesnila[6,8], Wolfgang F. Graier[1,9], and Roland Malli[1,9*]

[1] Gottfried Schatz Research Center, Molecular Biology and Biochemistry, Medical University of Graz, Neue Stiftingtalstraße 6/6, 8010 Graz, Austria
[2] Department of Pharmacology, Toxicology and Clinical Pharmacy, Institute of Pharmacy, Eberhard Karls University of Tuebingen, Auf der Morgenstelle 8, 72076 Tuebingen, Germany
[3] Natural and Medical Sciences Institute, University of Tuebingen, 72770 Reutlingen, Germany
[4] Gottfried Schatz Research Center, Biophysics, Medical University of Graz, Neue Stiftingtalstraße 6/6, 8010 Graz, Austria
[5] Department of Neurosurgery, Medical University of Graz, Auenbruggerplatz 29, 8036 Graz, Austria
[6] Laboratory of Experimental Stroke Research, Institute for Stroke and Dementia Research, University of Munich Medical Center, 81377 Munich, Germany
[7] Institute of Physiology I, Medical Faculty, University of Bonn, 53127 Bonn, Germany Munich
[8] Cluster for Systems Neurology (SyNergy), Munich, Germany
[9] BioTechMed Graz, Mozartgasse 12/II, 8010 Graz, Austria
* Correspondence to roland.malli@medunigraz.at

The supplementary information includes the following chapters

1) **Disclosure**
2) **Experimental Methods:**
   - Substances
   - Protein Purification Buffers
   - Protein Purification
   - Buffers for *In Vitro* Fluorimetry
   - Cell Culture and Transfection
   - Buffers for Fluorescence Microscopy
   - Fluorescence Microscopy
   - Western Blot Analysis
   - Glutamate Addition to Primary Rat Neurons
   - Data Processing and Statistical Analysis
3) **Tables & Figures:**
   - **Table S-1:** List of all plasmids/vectors used for mammalian and bacterial expression
   - **Table S-2:** Sequences of targeting signals and constructs used
   - **Table S-3:** Overview of the microscopes used for measuring GEPII and pH-Lemon (fusion) constructs
   - **Table S-4:** Comparison of targeted and immobilized pH-Lemon and GEPII 1.0 variants
   - **Figure S-1:** GPI-Targeting of Biosensors Leads to Sensor Accumulation within the Secretory Pathway
   - **Figure S-2:** Biosensor Localization within the Secretory Pathway Impacts Sensor Functionality, Possibly Due to Glycosylation
   - **Figure S-3:** Characterization of TAv-fused Sensors in Solution
   - **Figure S-4:** Constructs and Scheme for the Immobilization of TAv-fused Biosensors

Figure S-5: Functionality of TAv-pH-Lemon and TAv-GEPII 1.0 after Immobilization
Figure S-6: Local Immobilization of TAv-sensors Using Microperfusion
Figure S-7: Visualization of glutamate-induced K^+ efflux from primary neurons using surface-coupled TAv–GEPII 1.0.
Figure S-8: Cell & Sensor Preparation and Application to Living Cells

1) Disclosure

Parts of the methods & results presented in this paper are part of the dissertation "Visualization of local intracellular & cell surface cation alterations using fluorescent protein-based probes" [1] submitted in 04/2020 by Sandra Burgstaller at the Medical University of Graz, Department of Molecular Biology and Biochemistry (Graz, Austria). The full text will be available online after 04/2022 at the homepage of the Medical University of Graz https://online.medunigraz.at/mug_online/wbAbs.showThesis?pThesisNr=58221&pOrgNr=1

2) Experimental Methods

Substances. Substances purchased from Carl Roth (Graz, Austria): agar–agar Kobe I, agarose, bacterial protease inhibitor cocktail, $CaCl_2$, D-glucose, glutamate, HCl (30%), HEPES, KCl, KH_2PO_4, MES, $MgCl_2$, MOPS, NaCl, NaH_2PO_4, NaOH, Triton X-100, trypton/pepton, and yeast extract. Substances purchased from Sigma Aldrich (Vienna, Austria): benzonase nuclease, biotin, Dulbecco´s Modified Eagle's Medium (DMEM), gramicidin, imidazole, isopropyl-beta-D-thiogalactoside (IPTG), and N-methyl-D-glucamine (NMDG). Substances purchased from ThermoFisher Scientific (Vienna, Austria): fetal calf serum (FCS), fungizone, Gibco RPMI 1640 cell culture medium, MEM Glutamax supplement, MEM amino acids, penicillin-streptomycin, and protino Ni-NTA agarose.

Protein purification buffers. Lysis buffer (in mM): 150 NaCl, 100 Na_2HPO_4, 10 imidazole, 250 units benzonase nuclease, 100 µl of bacterial protease inhibitor cocktail (diluted according to the manufacturer's protocol), pH 8.0. Washing buffers for Ni-NTA purification: i) washing buffer 1 (in mM) 150 NaCl, 100 Na_2HPO_4, 20 imidazole, pH 8.0; ii) washing buffer 2 (in mM) 1000 NaCl, 100 Na_2HPO_4, 20 imidazole, pH 8.0. Purification buffer for elution from Ni-NTA columns (in mM): 150 NaCl, Na_2HPO_4, 300 imidazole, pH 8.0. Recombinant protein buffer (RPB) buffer used for desalting and storage of purified proteins: 10 HEPES, pH adjusted to 7.3 with N-Methyl-D-Glucamine (NMDG).

Protein purification. For the purification of TAv-constructs, TAv-pH-Lemon and TAv-GEPII 1.0 were transformed into chemically competent one shot BL21 star (DE3) *E.coli* (ThermoFisher Scientific). A standard protocol was used for transformation according to the manufacturer´s guidelines. One single colony was picked from the LB-agar plate, transferred into 10 mL of LB medium containing 50 µg/mL kanamycin and incubated over-night. The next day, the pre-culture was added to 1 L LB-medium containing kanamycin and incubated at 37°C on a shaker (MaxQ 8000 HP Incubated & Refrigerated Shaker, ThermoFisher Scientific) until an OD600 of 0.6-0.8 was reached. TAv-pH-Lemon and TAv-GEPII 1.0 expression was induced by the addition of 1 mM IPTG and the culture was cultivated at 20°C overnight in the dark.

Cells were harvested using centrifugation at 4°C and 6000 rpm (Sorvall LYNX 6000, ThermoFisher Scientific) and the pellet was resuspended in 15 mL of lysis buffer (see protein purification buffers) containing protease inhibitor cocktail. Sonication (QSONICA Ultrasonic Processor; 12 min, 50% amplitude, 1 sec on/off) was used to break up the cells. After additional centrifugation for 45 min at 10.000 rpm and 4°C, the supernatant was collected and applied onto gravity-based Ni-NTA (nickel affinity) columns with a flow rate of approximately1-2 mL/minute. After application of 15 mL of lysis buffer, 15 mL of washing buffer 1 and 15 mL of washing buffer 2 were applied (see protein purification buffers). The bound protein was again washed with 15 mL of washing buffer 1, followed by the application of 10 mL of purification buffer for elution (see protein purification buffers). Volume reduction of the protein solution was reached by centrifugation at 3.600 rpm at 4°C (Eppendorf centrifuge 5810 R, Eppendorf, Vienna, Austria) in concentrator tubes with a molecular cut-off of 30 kDa (Amicon Ultra-15, Merck Chemicals, and Life Science, Vienna, Austria). The centrifugation time was adjusted to reach a final volume of 1-0.5 ml protein solution. The protein solution was further purified and desalted using Cytiva PD MidiTrapsephadex columns (Fisher Scientific). The protein concentration was determined using NanoDrop 1000 UV/VIS spectrometer (ThermoFisher Scientific).

Buffers for *in vitro* fluorimetry. TAv-pH-Lemon was diluted with RPB buffers with differently adjusted pH values ranging from 2.0 to 9.0. For the adjustment of pH values MES (pH <5.0), HEPES (pH 5.5-9.0) and MOPS (pH >9.0) were used. TAv-GEPII 1.0 was diluted with RPB buffers (pH 7.3) containing different concentrations of KCl.

Plasmids and Targeting. TAv–pH-Lemon and TAv–GEPII 1.0 constructs were purchased from Gene Universal (Newark, DE, USA). ER-BirA-mCherry and AviTag-mCherry-GPI plasmids in a pcDNA3.1(-) vector backbone were purchased from Gene Universal. An adenovirus 5 (AV5) vector encoding ER-BirA and AviTag-mCherry-GPI was purchased from VectorBuilder (Neu-Isenburg, Germany). AviTag-mCherry-GPI (open reading frame 1; ORF1) and ER-BirA (ORF2) were separated by an internal ribosomal entry site (IRES). Endoplasmic reticulum (ER)–GEPII 1.0 and GEPII 1.0-GPI were generated by conventional PCR-, restriction-, and ligation-based cloning. ER targeting was achieved by fusing the calreticulin-targeting sequence to the N-terminus of GEPII 1.0 or ER-BirA-mCherry in combination with the ER retention signal KDEL on the C-terminal end. To target pH-Lemon, GEPII 1.0, or AviTag-mCherry-GPI at the cell surface, the targeting sequences of CDH13 were used. An overview of the biosensors used can be found in Table S-1. Detailed sequences are displayed in Table S-2.

Cell Culture and Transfection. Cell culture materials were obtained from Greiner Bio-One (Kremsmünster, Austria). HeLa cells (ATCC, Wesel, Germany) and HeLa cells stably expressing pH-Lemon–GPI[2] were cultured in Dulbecco's modified Eagle's medium supplemented with 10% fetal calf serum, 1x penicillin-streptomycin, and 2.5-µg/mL fungizone (ThermoFisher Scientific, Vienne, Austria). Gibco RPMI 1640 medium (ThermoFisher Scientific) was used to cultivate INS-1 832/13 cells (provided by courtesy of C. B. Newgard, Department of Pharmacology and Cancer Biology, Duke University School of Medicine, NC, USA). For transfection of HeLa and INS-1 832/13 cells, PolyJet transfection reagent (SignaGen

Laboratories, Rockville, MD, USA) was used according to the manufacturer's instructions. The next day, the transfection mix was removed, and the cells were incubated for another 20 hours. For co-transfection of ER-BirA-mCherry and AviTag-mCherry-GPI constructs, the medium was supplemented with 10 µM of biotin. INS-1 832/13 cells and primary rat neurons were infected with AV5 at a MOI of 50–60 in a biotin-supplemented medium two days before measurements. The virus-containing medium was removed after 24 hours, and the cells were incubated for another 20 hours before measurements.

For the isolation of primary rat neurons, 4 cortical hemispheres were quickly removed from two P0-P1 rat pups (sacrificed by decapitation), washed with phosphate buffer, crosswise-chopped in 100 µm squares (McIlwain Tissue Chopper, Campden Instruments LTD, UK) and transferred to 1 mL Accutase (Thermo Fisher Scientific, USA) for 20 min at 37°C. Enzymatic digestion was stopped by addition of serum-containing medium. The suspension was then filtered through a 0.4 µm cell strainer and centrifuged for 5 min at 300 g. For cultivation of neurons, cells were re-suspended in cultivation medium (Neurobasal A Medium supplemented with 1 % B-27, 0.5 mM GlutaMAX (all Thermo Fisher Scientific, USA), 5 ng/mL β-FGF and 20 ng/mL EGF (both PrePro Tech, USA) and containing 0.2% Normocin (Invivogen, USA). After 4 days, β-FGF concentration was increased to 10 ng/mL.

Buffers for fluorescence microscopy. Cell equilibration buffer containing (in mM) 2 CaCl2, 10 D-glucose, 10 HEPES, 5 KCl, 2 L-glutamine, 0.44 KH_2PO_4, 1 $MgCl_2$, 135 NaCl, 2.6 $NaHCO_3$, 0.34 Na_2HPO_4, 1X MEM, pH 7.4. A physiological buffer containing (in mM) 138 NaCl, 10 D-Glucose, 10 HEPES, 5 KCl, 2 $CaCl_2$, 1 $MgCl_2$ with a pH 7.4 was used for perfusion. For pH measurements, physiological buffers with different pH values were used, for measuring K^+ levels, a physiological buffer with different K^+ concentrations [K^+] were used. KCl was added to the buffer, while the osmolarity was retained by a respective decrease of NaCl. For intracellular K^+ titrations in permeabilized cells, a physiological buffer containing 15 µM Gramicidin was used. For washing steps, phosphate-buffered saline (PBS) was used.

Fluorescence Microscopy. Before cyto-GEPII 1.0 measurements, the cells were equilibrated for at least 30 minutes in cell equilibration buffer, while cells for immobilization experiments were washed with PBS prior to immobilization. Time-lapse imaging experiments of cells were performed on different microscopes. First, an Olympus IX73 inverted microscope (Olympus, Vienna, Austria) with a 40x objective (UApo N 340, 40X/1.35 Oil, ∞/0.17/FN22, Olympus) or a 10x objective (UPLXAPO10X, 10x/0.4 Air, ∞/0.17) was sued. The microscope was equipped with a 455 nm and a 530 nm LED light source (OMICRON electronics, Vienna, Austria), with 427/10 and 575/15 excitation filters (AHF Analysentechnik, Tübingen, Germany) and a Retiga R1 CCD-camera (TELEDYNE QIMAGING, Surrey, Canada). Second, an iMic inverted and advanced fluorescent microscope (TILL Photonics, Gräfelfing, Germany) with a motorized sample stage and a 40x objective (Zeiss) connected to a Polychrome V light source was used. Images were captured using an AVT Stringray 25 F145B (Allied Vision Technologies, Stadtroda, Germany). Third, a Zeiss Axio Observer Z1 (Zeiss, Oberkochen, Germany) equipped with a 40x objective (ObjectivFluar, 40X/1,30 Oil M27, Zeiss), and a 455 nm as well as a 505-600 nm LED light source (OMICRON electronics) was used. The system was equipped with 427/10 and 575/15 excitation filters (AHF Analysentechnik) and a pco.panda 4.2

bi sCMOS-camera (PCO, Kelheim, Germany). Emission of FRET-sensors was detected at 475 and 530 nm, respectively, and emissions were either separated using a photometrics DV2 image splitter (Teledyne Technologies Inc., Arizona, US), the TILL Dichrotome (TILL Photonics), or an Optosplit II image splitter (Teledyne Technologies Inc.). Microscope control and the acquisition was either performed using VisiViewPremier Acquisition software (Visitron Systems, Puchheim, Germany) or the live acquisition (LA) software (TILL Photonics). All microscopes were connected to a gravity-based perfusion system (NGFI, Graz, Austria; www.ngfi.eu). The 30 mm glass slide (with cells growing on top) was inserted into a perfusion chamber (NGFI), which was connected to the perfusion system and a vacuum pump to ensure a laminar flow.

For high-resolution imaging, a Nipkow spinning disk-based array confocal laser scanning microscope (ACLSM), which consisted of a Zeiss Axiovert 200M (Zeiss) was used. The microscope was equipped with a 100x objective (α Plan-Fluar100X/1.45 oil objective, Zeiss) and with VoxCell Scan (VisiTech, Sunderland, UK). An air-cooled argon-ion laser system (series 543, CVI Melles Griot, CA, USA) was used as the light source. For imaging, the emission filters ET480/40m (CFP) and ET525/50m (EYFP) (Chroma Technologies, VT, USA) were used. CFP and YFP were excited using either 445 nm or 510 nm laser light, respectively and RFP was excited at 561 nm. Emissions at 475 nm, 525 nm, and 610 nm (CFP, YFP, or mCherry) were collected by a CCD camera (CoolSnap HQ2, Photometrics, Tucson, Arizona, USA) and acquire at a binning of 1, 2, or 4. Microscope control and acquisition was performed using VisiView Premier Acquisition software (Visitron Systems, Puchheim, Germany). A summary of the microscopes used can be found in Table S-3.

Western Blot Analysis. HEK293 cells expressing either cytosolic GEPII 1.0 or membrane targeted GEPII 1.0-GPI were treated with or without PNGaseF and cell extracts were loaded onto a 7.5% SDS-PAGE. Anti-GFP Living Colors (clone JL-8, Takara)) was used (1:3000 dilution) and incubated at 4°C over night. HRP-conjugated Goat Anti-mouse IgG (Jackson ImmunoResearch, West Grove, United States) (1:10.000 dilution) was incubated at room temperature for 1 hour. For detection, PierceTMECL Western Blotting Substrate (Thermo Scientific) and Odyssey Fc Imaging System (Li-Cor, Lincoln, Nebraska, United States) were used.

Glutamate Addition to Primary Rat Neurons. Glutamate was injected into a buffer with 0 mM K^+ to reach a final concentration of 500 µM (first injection) and 1mM (second injection), respectively, while imaging. The perfusion system and the pump were stopped before injection to prevent removal of glutamate due to perfusion. To test the reversibility of the effect, the perfusion was started and a buffer with 140 mM K^+, followed by 0 mM K^+ was perfused.

Data Processing and Statistical Analysis. The data were analyzed using Microsoft Office Excel (Microsoft, Redmond, WA, USA), MetaMorph (Molecular Devices, San Jose, CA, USA), ImageJ (National Institutes of Health, Bethesda, MD, USA), and GraphPad Prism 5 (GraphPad Software, San Diego, CA, USA). GraphPad Prism 5 (GraphPad Software) and CorelDraw (Corel Corporation, Ottawa, ON, Canada) were used for data visualization. Phyre2 (Structural Bioinformatics Group, Imperial College, London, UK) and PyMOL (Schrödinger, New York, NY, USA) were used for 3D prediction and visualization. The NetNGlyc 1.0 server

(Department of Bio and Health Informatics, Technical University of Denmark, Kongens Lyngby, Denmark) was used to predict possible glycosylation sites. Statistical tests were performed using GraphPad Prism 5. To fit the data for the EC_{50} values, all measurements were plotted in Prism 5. By using the sigmoidal dose-response (variable slope) equation in Prism, the EC_{50} values and the 95% confidence interval could be determined.

For the analysis of the plasma membrane versus intracellular fluorescence, ImageJ was used for the image analysis as followed: the CFP image was added on top of the FRET image (to create the sum of both images) to measure every fluorescence signal from the cell. Then the background was subtracted as determined in one ROI with no cells. The image was duplicated (image 1 and image 2) and one ROI was drawn at the outside of the plasma membrane to measure the sum of intensities in image 1 (i.e. whole cell fluorescence). We then drew one ROI on the inside of the plasma membrane on image 2 and cut out the region containing all intracellular structures (this created an intracellular fluorescence intensity of zero). The region from image 1 (= extracellular region) was copied into image 2 and the intensity was measured (i.e. membrane only). To generate the intracellular fluorescence the membrane values were subtracted from the whole cell values. One confocal plane was analyzed with a special focus on image acquisition in the middle of the cell (i.e. to image through the nucleus). For all measurements of GEPII 1.0 constructs (cyto, ER and GPI), TAv-GEPII 1.0 and TAv-pH-Lemon constructs, the whole cell was taken as one ROI. The ROI was therefore drawn at the outer plasma membrane. The background ROI was selected in a region with no cells and subtracted for final analysis.

3) Tables & Figures

Table S-1: List of all plasmids/vectors used for mammalian and bacterial expression.

Sensor	*Details*	*Reference*
**GEPII 1.0-GPI**	Cell-surface targeted K^+ sensor	This work
**Cytosolic GEPII 1.0**[3]	Cytosolic K^+ sensor	Source: Bischof et al., Nat. Commun, 2017
**ER-GEPII 1.0**	ER-targeted K^+ sensor	This work
**pH-Lemon-GPI**[4]	Cell-surface targeted pH sensor	Source: Burgstaller et al., ACS Sensors, 2019
**TAv-pH-Lemon**	pH sensor for bacterial expression fused to Traptavidin (TAv)	This work
**TAv-GEPII 1.0**	K^+ sensor for bacterial expression fused to Traptavidin (TAv)	This work
**ER-BirA-mCherry**	ER-targeted mCherry-fused biotin ligase	This work
**AviTag-mCherry-GPI**	Cell-surface targeted AviTag tagged with mCherry	This work
**Adenovirus type 5 (ER-BirA+AviTag-mCherry-GPI)**	Encoding for ER-BirA + AviTag-mCherry-GPI	This work

Table S-2: Detailed sequences of targeting signals and constructs used

GPI-anchor leader signal (N-terminal) + GPI-anchor attachment signal (C-terminal)
ATGCAGCCGAGAACTCCGCTCGTTCTGTGCGTTCTCCTGTCCCAGGTGCTGCTGCTAACAT
CTGCAGGATCC +
GACTGCAACGCGGCAGGGGCCCTGCGCTTCAGCCTGCCCTCAGTCCTGCTCCTCAGCCTC
TTCAGCTTAGCTTGTCTGT

ER-targeting sequence (Calrecticulin, N-terminal) + KDEL (C-terminal)
ATGCTGCTGCCCGTCCCCCTGCTGCTGGGCCTGCTGGGCGCCGCCGCCGA +
AAGGACGAGCTG

Cytosolic targeting sequence (=nuclear exporting sequence, C-terminal):
TTGCCTCCATTAGAACGATTGACGTTA

TAv-pH-Lemon (mTurquoise2-Linker-EYFP):
ATGGCTGAAGCTGGTATCACCGGCACCTGGTACAACCAGCTGGGATCAACCTTCATCGTT
ACCGCTGGTGCTGACGGTGCTCTGACCGGTACGTACGAATCCGCTGTTGGTAACGCTGAA
GGCGATTACGTTCTGACCGGTCGTTACGACTCCGCTCCGGCTACCGACGGTTCCGGAACC
GCTCTGGGTTGGACCGTTGCTTGGAAAAACAACTACCGTAACGCTCACTCCGCTACCACC
TGGTCTGGCCAGTACGTTGGTGGTGCTGAAGCTCGTATCAACACCCAGTGGTTGTTGACC
TCCGGCACCACCGAAGCTAACGCGTGGAAATCCACCCTGGTTGGTCACGACACCTTCACC
AAAGTTAAACCGTCCGCTGCTTCCGGCGGAGGTGGGTCCGGCGGAGGTGGGTCCGGCGG
AGGTGGGTCCGCCTCGAGTATGGTGAGCAAGGGCGAGGAGCTGTTCACCGGGGTGGTGC
CCATCCTGGTCGAGCTGGACGGCGACGTAAACGGCCACAAGTTCAGCGTGTCCGGCGAG
GGCGAGGGCGATGCCACCTACGGCAAGCTGACCCTGAAGTTCATCTGCACCACCGGCAA
GCTGCCCGTGCCCTGGCCCACCCTCGTGACCACCCTGTCCTGGGGCGTGCAGTGCTTCGCC
CGCTACCCCGACCACATGAAGCAGCACGACTTCTTCAAGTCCGCCATGCCCGAAGGCTAC
GTCCAGGAGCGCACCATCTTCTTCAAGGACGACGGCAACTACAAGACCCGCGCCGAGGT
GAAGTTCGAGGGCGACACCCTGGTGAACCGCATCGAGCTGAAGGGCATCGACTTCAAGG
AGGACGGCAACATCCTGGGGCACAAGCTGGAGTACAACTACTTTAGCGACAACGTCTAT
ATCACCGCCGACAAGCAGAAGAACGGCATCAAGGCCAACTTCAAGATCCGCCACAACAT
CGAGGACGGCGGCGTGCAGCTCGCCGACCACTACCAGCAGAACACCCCCATCGGCGACG
GCCCCGTGCTGCTGCCCGACAACCACTACCTGAGCACCCAGTCCAAGCTGAGTAAAGACC
CCAACGAGAAGCGCGATCACATGGTCCTGCTGGAGTTCGTGACCGCCGCCGGGATCACTC
TCGGCATGGACGAGCTGTACAAGGGTGGAGGCGGTAGCGAATTCATGGTGAGCAAGGGC
GAGGAGCTGTTCACCGGGGTGGTGCCCATCCTGGTCGAGCTGGACGGCGACGTAAACGG
CCACAAGTTCAGCGTGTCCGGCGAGGGCGAGGGCGATGCCACCTACGGCAAGCTGACCC
TGAAGTTCATCTGCACCACCGGCAAGCTGCCCGTGCCCTGGCCCACCCTCGTGACCACCT
TCGGCTACGGCCTGCAGTGCTTCGCCCGCTACCCCGACCACATGAAGCAGCACGACTTCT
TCAAGTCCGCCATGCCCGAAGGCTACGTCCAGGAGCGCACCATCTTCTTCAAGGACGACG
GCAACTACAAGACCCGCGCCGAGGTGAAGTTCGAGGGCGACACCCTGGTGAACCGCATC
GAGCTGAAGGGCATCGACTTCAAGGAGGACGGCAACATCCTGGGGCACAAGCTGGAGTA
CAACTACAACAGCCACAACGTCTATATCATGGCCGACAAGCAGAAGAACGGCATCAAGG
TGAACTTCAAGATCCGCCACAACATCGAGGACGGCAGCGTGCAGCTCGCCGACCACTACC
AGCAGAACACCCCCATCGGCGACGGCCCCGTGCTGCTGCCCGACAACCACTACCTGAGCT
ACCAGTCCGCCCTGAGCAAAGACCCCAACGAGAAGCGCGATCACATGGTCCTGCTGGAG
TTCGTGACCGCCGCCGGGATCACTCTCGGCATGGACGAGCTGTACAAGGGTACCCACCAC
CACCACCACCACTGAGCTGAGC

TAv-GEPII 1.0 (mseCFP-Kbp-cpV):

ATGGCTGAAGCTGGTATCACCGGCACCTGGTACAACCAGCTGGGATCaACCTTCATCGTT
ACCGCTGGTGCTGACGGTGCTCTGACCGGTACgTACGAATCCGCTGTTGGTAACGCTGAA
GGCGATTACGTTCTGACCGGTCGTTACGACTCCGCTCCGGCTACCGACGGTTCCGGAACC
GCTCTGGGTTGGACCGTTGCTTGGAAAAACAACTACCGTAACGCTCACTCCGCTACCACC
TGGTCTGGCCAGTACGTTGGTGGTGCTGAAGCTCGTATCAACACCCAGTGGTTGTTGACC
TCCGGCACCACCGAAGCTAACGCGTGGAAATCCACCCTGGTTGGTCACGACACCTTCACC
AAAGTTAAACCGTCCGCTGCTTCCGGCGGAGGTGGGTCCGGCGGAGGTGGGTCCGGCGG
AGGTGGGTCCGGCGGAGGTGGGTCCGGCGGAGGTGGGTCCGGCGGAGGTGGGTCCGCCT
CGAGTATGGTGAGCAAGGGCGAGGAGCTGTTCACCGGGGTGGTGCCCATCCTGGTCGAG
CTGGACGGCGACGTAAACGGCCACAGGTTCAGCGTGTCCGGCGAGGGCGAGGGCGATGC
CACCTACGGCAAGCTGACCCTGAAGTTCATCTGCACCACCGGCAAGCTGCCCGTGCCCTG
GCCCACCCTCGTGACCACCCTGACCTGGGGCGTGCAGTGCTTCGCCCGCTACCCCGACCA
CATGAAGCAGCACGACTTCTTCAAGTCCGCCATGCCCGAAGGCTACGTCCAGGAGCGTAC
CATCTTCTTCAAGGACGACGGCAACTACAAGACCCGCGCCGAGGTGAAGTTCGAGGGCG
ACACCCTGGTGAACCGCATCGAGCTGAAGGGCATCGACTTCAAGGAGGACGGCAACATC
CTGGGGCACAAGCTGGAGTACAACTACATCAGCCACAACGTCTATATCACCGCCGACAA
GCAGAAGAACGGCATCAAGGCCCACTTCAAGATCCGCCACAACATCGAGGACGGCGGCG
TGCAGCTCGCCGACCACTACCAGCAGAACACCCCCATCGGCGACGGCCCCGTGCTGCTGC
CCGACAACCACTACCTGAGCACCCAGTCCAAGCTGAGCAAAGACCCCAACGAGAAGCGC
GATCACATGGTCCTGCTGGAGTTCGTGACCGCCGCCATCGATATGGGTCTGTTCAATTTG
TGAAAGATGCCGGAGAAAAACTCTGGGACGCGGTTACAGGTCAGCACGATAAAGACGAT
CAGGCGAAGAAGGTGCAGGAGCATCTGAACAAAACCGGTATACCGGATGCCGATAAAGT
GAATATTCAAATTGCCGACGGCAAAGCGACGGTCACTGGTGACGGCCTGAGTCAGGAGG
CGAAGGAGAAAATCCTTGTTGCGGTGGGGAATATTTCCGGTATTGCCAGTGTCGATGATC
AGGTGAAAACGGCGACACCAGCCACTGCCAGCCAGTTTTATACCGTTAAGTCTGGCGACA
CTCTGAGTGCCATTTCCAAACAGGTCTACGGTAACGCTAATCTGTACAATAAAATCTTCG
AAGCGAATAAACCGATGCTAAAAAGCCCGGATAAAATTTATCCGGGGCAAGTGTTGCGT
ATTCCGGAAGAGGAATTCATGGACGGCGGCGTGCAGCTCGCCGACCACTACCAGCAGAA
CACCCCCATCGGCGACGGCCCCGTGCTGCTGCCCGACAACCACTACCTGAGCTACCAGTC
CAAGCTGAGCAAAGACCCCAACGAGAAGCGCGATCACATGGTCCTGCTGGAGTTCGTGA
CCGCCGCCGGGATCACTCTCGGCATGGACGAGCTGTACAAGGGTGGCAGCGGTGGCATG
GTGAGCAAGGGCGAGGAGCTGTTCACCGGGGTGGTGCCCATCCTGGTCGAGCTGGACGG
CGACGTAAACGGCCACAAGTTCAGCGTGTCCGGCGAGGGCGAGGGCGATGCCACCTACG
GCAAGCTGACCCTGAAGCTGATCTGCACCACCGGCAAGCTGCCCGTGCCCTGGCCCACCC
TCGTGACCACCCTGGGCTACGGCCTGCAGTGCTTCGCCCGCTACCCCGACCACATGAAGC
AGCACGACTTCTTCAAGTCCGCCATGCCCGAAGGCTACGTCCAGGAGCGCACCATCTTCT
TCAAGGACGACGGCAACTACAAGACCCGCGCCGAGGTGAAGTTCGAGGGCGACACCCTG
GTGAACCGCATCGAGCTGAAGGGCATCGACTTCAAGGAGGACGGCAACATCCTGGGGCA
CAAGCTGGAGTACAACTACAACAGCCACAACGTCTATATCACCGCCGACAAGCAGAAGA
ACGGCATCAAGGCCAACTTCAAGATCCGCCACAACATCGAGGGTACCCACCACCACCAC
CACCACTGAGCTGAGC

ER-BirA-mCherry:

GCTAGCATGCTGCTGCCCGTCCCCCTGCTGCTGGGCCTGCTGGGCGCCGCCGCCGACTCG
AGTATGAAGGATAACACCGTGCCACTGAAATTGATCGCCCTGTTAGCGAATGGCGAATTT
CACTCTGGCGAGCAGTTGGGTGAAACGCTGGGAATGAGCCGGGCGGCTATTAATAAACA
CATTCAGACACTGCGTGATTGGGGCGTTGATGTATTTACTGTTCCGGGTAAAGGATACAG
CTTGCCCGAGCCAATCCAGTTACTTAATGCCGAAAAGATATTGAGTCAGCTGGATGACGG

CAGTGTAGCCGTGCTGCCAGTTATTGACTCCACGAATCAGTATCTCCTGGACCGCATCGG
AGAGCTTAAATCTGGCGATGCCTGTGTTGCAGAATATCAGCATGCTGGCCGTGGTCGTCG
TGGTCGGAAATGGTTTTCGCCTTTTGGCGCGAACGTATATTTGTCGATGTTCTGGCGTCTG
GAACAGGGCCCGGCAGCGGCGATTGGTTTAAGTCTGGTTATCGGTATCGTGATGGCGGAA
GTATTACGCAAGCTGGGTGCAGATAAAGTTCGTGTTAAATGGCCTAATGACCTCTATCTG
CAGGATCGCAAGCTGGCAGGCATTCTTGTGGAGCTGACTGGCAAAACCGGCGATGCGGC
GCAAATAGTCATTGGTGCAGGGATCAACATGGCAATGCGTCGTGTTGAAGAGAGTGTCGT
TAATCAGGGGTGGATCACGCTGCAGGAAGCTGGGATCAATCTCGATCGTAATACTCTTGC
GGCCATGCTGATACGTGAATTACGCGCGGCGCTGGAACTCTTCGAGCAAGAAGGATTGGC
ACCTTATCTTTCGCGCTGGGAAAAGTTGGATAATTTTATTAATCGCCCAGTGAAACTTATC
ATTGGTGATAAAGAAATATTTGGCATTTCACGCGGAATAGACAAACAGGGTGCTTTATTA
CTTGAGCAGGATGGAATAATAAAACCCTGGATGGGCGGTGAAATATCCCTGCGTAGTGC
AGAAAAAAAGCTTATGGTGAGCAAGGGCGAGGAGGATAACATGGCCATCATCAAGGAGT
TCATGCGCTTCAAGGTGCACATGGAGGGCTCCGTGAACGGCCACGAGTTCGAGATCGAG
GGCGAGGGCGAGGGCCGCCCCTACGAGGGCACCCAGACCGCCAAGCTGAAGGTGACCAA
GGGTGGCCCCCTGCCCTTCGCCTGGGACATCCTGTCCCCTCAGTTCATGTACGGCTCCAAG
GCCTACGTGAAGCACCCCGCCGACATCCCCGACTACTTGAAGCTGTCCTTCCCCGAGGGC
TTCAAGTGGGAGCGCGTGATGAACTTCGAGGACGGCGGCGTGGTGACCGTGACCCAGGA
CTCCTCCCTGCAGGACGGCGAGTTCATCTACAAGGTGAAGCTGCGCGGCACCAACTTCCC
CTCCGACGGCCCCGTAATGCAGAAGAAGACCATGGGCTGGGAGGCCTCCTCCGAGCGGA
TGTACCCCGAGGACGGCGCCCTGAAGGGCGAGATCAAGCAGAGGCTGAAGCTGAAGGAC
GGCGGCCACTACGACGCTGAGGTCAAGACCACCTACAAGGCCAAGAAGCCCGTGCAGCT
GCCCGGCGCCTACAACGTCAACATCAAGTTGGACATCACCTCCCACAACGAGGACTACAC
CATCGTGGAACAGTACGAACGCGCCGAGGGCCGCCACTCCACCGGCGGCATGGACGAGC
TGTACAAGAAGCTTAAAGACGAGCTGTAA

AviTag-mCherry-GPI:
TCTAGAATGCAGCCGAGAACTCCGCTCGTTCTGTGCGTTCTCCTGTCCCAGGTGCTGCTGC
TAACATCTGCAGGATCCCTCGAGGGGTTGAATGATATTTTCGAAGCACAGAAAATTGAAT
GGCATGAGAAGCTTATGGTGAGCAAGGGCGAGGAGGATAACATGGCCATCATCAAGGAG
TTCATGCGCTTCAAGGTGCACATGGAGGGCTCCGTGAACGGCCACGAGTTCGAGATCGAG
GGCGAGGGCGAGGGCCGCCCCTACGAGGGCACCCAGACCGCCAAGCTGAAGGTGACCAA
GGGTGGCCCCCTGCCCTTCGCCTGGGACATCCTGTCCCCTCAGTTCATGTACGGCTCCAAG
GCCTACGTGAAGCACCCCGCCGACATCCCCGACTACTTGAAGCTGTCCTTCCCCGAGGGC
TTCAAGTGGGAGCGCGTGATGAACTTCGAGGACGGCGGCGTGGTGACCGTGACCCAGGA
CTCCTCCCTGCAGGACGGCGAGTTCATCTACAAGGTGAAGCTGCGCGGCACCAACTTCCC
CTCCGACGGCCCCGTAATGCAGAAGAAGACCATGGGCTGGGAGGCCTCCTCCGAGCGGA
TGTACCCCGAGGACGGCGCCCTGAAGGGCGAGATCAAGCAGAGGCTGAAGCTGAAGGAC
GGCGGCCACTACGACGCTGAGGTCAAGACCACCTACAAGGCCAAGAAGCCCGTGCAGCT
GCCCGGCGCCTACAACGTCAACATCAAGTTGGACATCACCTCCCACAACGAGGACTACAC
CATCGTGGAACAGTACGAACGCGCCGAGGGCCGCCACTCCACCGGCGGCATGGACGAGC
TGTACAAGAAGCTTGACTGCAACGCGGCAGGGGCCCTGCGCTTCAGCCTGCCCTCAGTCC
TGCTCCTCAGCCTCTTCAGCTTAGCTTGTCTGTAACTTAAG

IRES (as part of the AV5 vector {ER-BirA-mCherry – IRES - AviTag-mCherry-GPI}):
GCCCCTCTCCCTCCCCCCCCCCTAACGTTACTGGCCGAAGCCGCTTGGAATAAGGCCGGT
GTGCGTTTGTCTATATGTTATTTTCCACCATATTGCCGTCTTTTGGCAATGTGAGGGCCCG
GAAACCTGGCCCTGTCTTCTTGACGAGCATTCCTAGGGGTCTTTCCCCTCTCGCCAAAGGA
ATGCAAGGTCTGTTGAATGTCGTGAAGGAAGCAGTTCCTCTGGAAGCTTCTTGAAGACAA

ACAACGTCTGTAGCGACCCTTTGCAGGCAGCGGAACCCCCCACCTGGCGACAGGTGCCTC
TGCGGCCAAAAGCCACGTGTATAAGATACACCTGCAAAGGCGGCACAACCCCAGTGCCA
CGTTGTGAGTTGGATAGTTGTGGAAAGAGTCAAATGGCTCTCCTCAAGCGTATTCAACAA
GGGGCTGAAGGATGCCCAGAAGGTACCCCATTGTATGGGATCTGATCTGGGGCCTCGGTG
CACATGCTTTACATGTGTTTAGTCGAGGTTAAAAAAACGTCTAGGCCCCCCGAACCACGG
GGACGTGGTTTTCCTTTGAAAAACACGATGATAATATGGCCACAACC

Table S-3: Overview of the microscopes used for measuring GEPII and pH-Lemon (fusion) constructs.

Microscope	Light source	Excitation Light	Excitation Filter	Emission Filter	Figure
Array confocal laser scanning microscope (ACLSM)	Diode lasers (Visitron Systems)	445 nm	AOTF* (Visitron Systems; Filter free)	ET480/40 (Chroma Technology Corporation, VT, USA)	F1c,e F3b-e FS-1 FS4a,b
		515 nm		ET535/30 (Chroma Technology Corporation)	
		561 nm		E570lpv2 (Chroma Technology Corporation)	
iMic (Till photonics)	Polychrom V	430 nm	CFP/YFP/mCherry ET Tripleband Exciter (AHF Analysentechnik, Tübingen, Germany)	CFP/YFP/mCherry ET Tripleband Emitter (AHF Analysentechnik)	F2b,d F4a-d
		505 nm			
		575 nm			
Olympus IX73	Omicron LEDHub High-Power LED Light Engine	455 nm	427/10 BrightLine HC (AHF Analysentechnik)	CFP/YFP/mCherry ET Tripleband Emitter (AHF Analysentechnik)	F4e,f F5a,b FS-6a-e FS7a,c,d,e
		505 nm	510/10 BrightLine HC (AHF Analysentechnik)		
		595 nm	575/15 BrightLine HC (AHF Analysentechnik)		
Zeiss Axio Observer Z1	Omicron LEDHub High-Power LED [illegible]	455 nm	427/10 BrightLine HC (AHF Analysentechnik)	475/543/701 HC Triple Filter (AHF Analysentechnik)	FS-2d-g

* AOTF: **A**cousto **O**ptic **T**unable **F**ilter

Table S-4: Comparison of targeted and immobilized pH-Lemon and GEPII 1.0 variants. All $[K^+]$ values are displayed in mM.

Sensor		in vitro	in situ	immobilized on cells	immobilized on glass
GEPII 1.0	EC_{50}	0.4 (0.4–0.5)*	0.8 (permeabilized with digitonin)*		
	EC_{50}		3.4 (3.2-3.7) (permeabilized with gramicidin)		
TAv-GEPII 1.0	EC_{50}	0.5 (0.5-0.6)		3.5 (2.6-4.6)	4.2 (1.8-9.5)
	dynamic range	116.4 ± 1.8		33.7 ± 2.7	31.2 ± 6.9
	basal	0.9 ± 0.007		4.1 ± 0.3	2.1 ± 0.4
pH-Lemon	EC_{50}	5.4 (5.3–5.5)#	6.3 (6.3–6.4)#		
TAv-pH-Lemon	EC_{50}	5.2 (5.0-5.4)		7.1 (7.0-7.2)	7.0 (6.9-7.2)
	dynamic range	4,315 ± 506.4		2,100 ± 250.7	504.2 ± 59.3
	basal	0.1 ± 0.01		0.3 ± 0.1	0.3 ± 0.1

* Source: Bischof et al., Nat. Commun., 2017
# Source: Burgstaller et al., ACS Sensors, 2019

Supplementary Figure 1

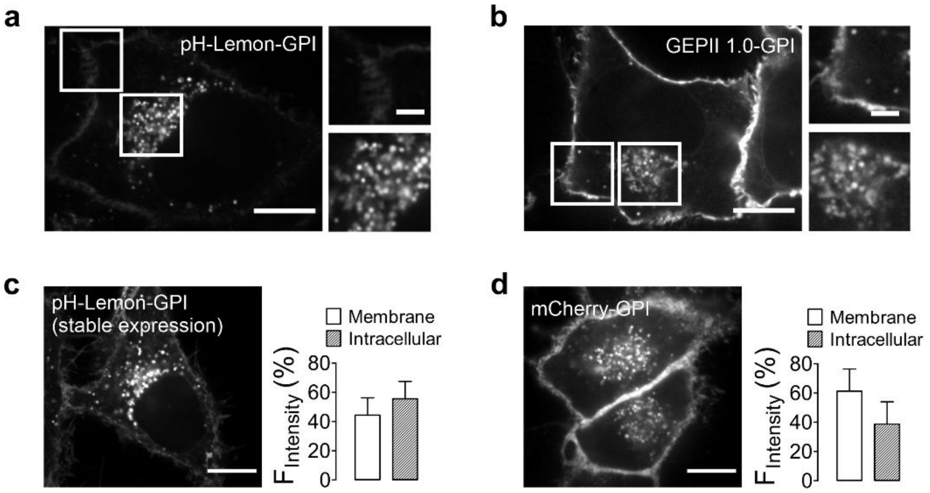

Figure S-1: GPI-Targeting of Biosensors Leads to Sensor Accumulation within the Secretory Pathway. (**a**) pH-Lemon-GPI or (**b**) GEPII 1.0-GPI with zooms on the plasma membrane (upper small images) and intracellular structures (lower small images) acquired using ACLSM. Shown is one z-plane. The scale bars represent 10 µm and 2 µm. (**c**) Representative ACLSM images (left) and statistical analysis of the fluorescence distribution of plasma membrane versus intracellular structures (right) of HeLa cells stably expressing pH-Lemon-GPI. The sum of mTurquoise2 and EYFP intensities from the membrane (white bar) and the intracellular structures (dashed bar) are shown. 16 cells were analyzed. Data represents average ± SD. (**d**) Representative ACLSM images and statistical analysis of the fluorescence distribution of plasma membrane versus intracellular structures of HeLa cells expressing a mCherry-GPI construct. The RFP intensities from the membrane (white bar) and the intracellular structures (dashed bar) are shown. 26 cells were analyzed. Data represents average ± SD.

Supplementary Figure 2

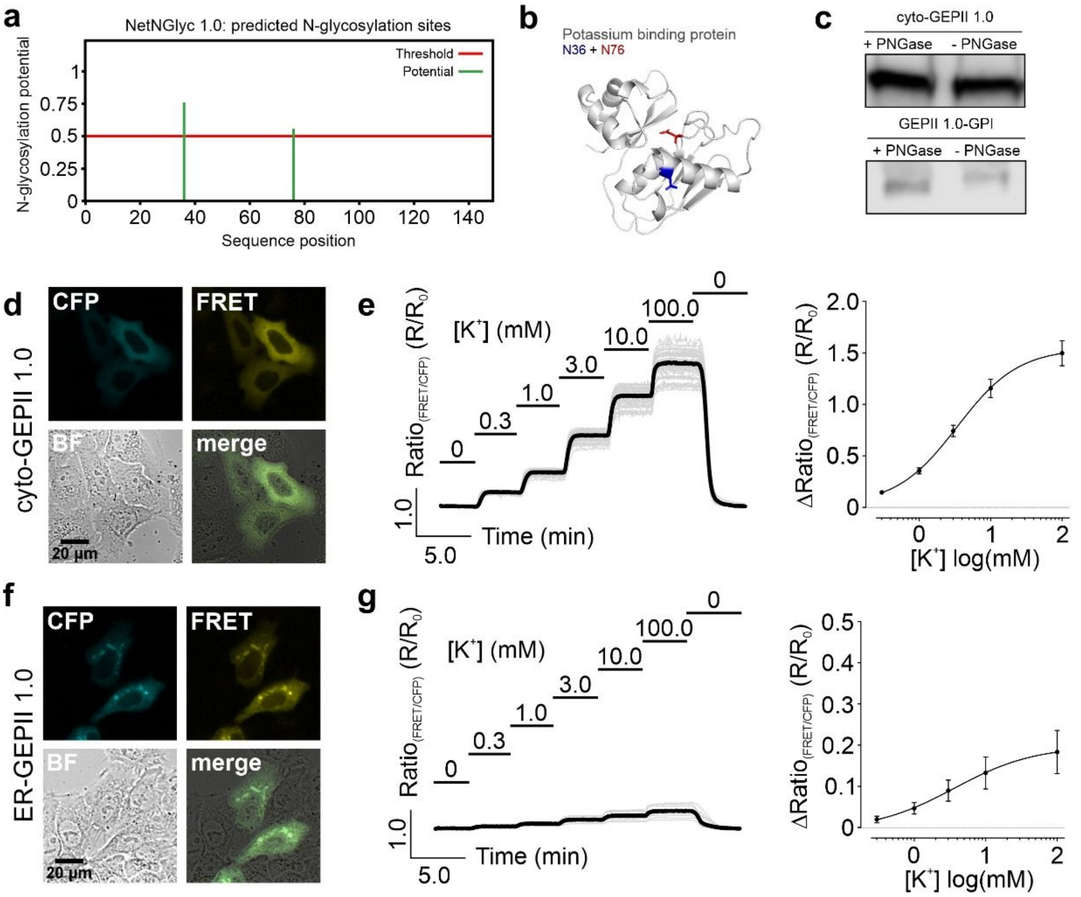

Figure S-2: Biosensor Localization within the Secretory Pathway Impacts Sensor Functionality, Possibly Due to Glycosylation. (**a**) Prediction of N-glycosylation sites using NetNGlyc1.0 revealed two possible glycosylation positions at amino acid positions N36 and N76 (methionine counted as amino acid 1) of the Kbp (green peaks). (**b**) 3D model of Kbp generated with Phyre2. The model was represented using PyMol Viewer and is represented as cartoon model (grey). N36 and N76 (methionine counted as amino acid 1) which represent potentially glycosylated amino acids as demonstrated in (**a**) are additionally shown as sticks and colored in blue (N36) and red (N76). (**c**) Western blot analysis (7.5% SDS-PAGE) of HEK293 cells expressing cytosolic GEPII and membrane targeted GPI-GEPII with (left lanes) and without (right lanes) PNGaseF incubation to cleavage N-glycans with the shift of molecular weight in GPI-GEPII proving N-glycosylation. (**d**) Fluorescence and brightfield images of HeLa cells expressing cytosolic GEPII 1.0. The scale bar represents 20 µm. (**e**) Average FRET-ratio signal (black line) and single-cell responses (grey lines) of cyto-GEPII 1.0 upon application of different K^+ buffers via a gravity-based perfusion system over time (left panel). Right panel displays the concentration-response curve with estimated EC_{50} values of cyto-GEPII 1.0. Data represent average ± SD, n=5. (**f**) Fluorescence and brightfield images of HeLa cells expressing ER-targeted GEPII 1.0. The scale bar represents 20 µm. (**g**) Average FRET-ratio signal (black line) and single-cell responses (grey lines) of ER-GEPII 1.0 upon application of different K+ buffers via a gravity-based perfusion system over time (left panel). The right panel displays the concentration-response curve with estimated EC_{50} values of ER-GEPII 1.0. Data represent average ± SD, n=4.

Supplementary Figure 3

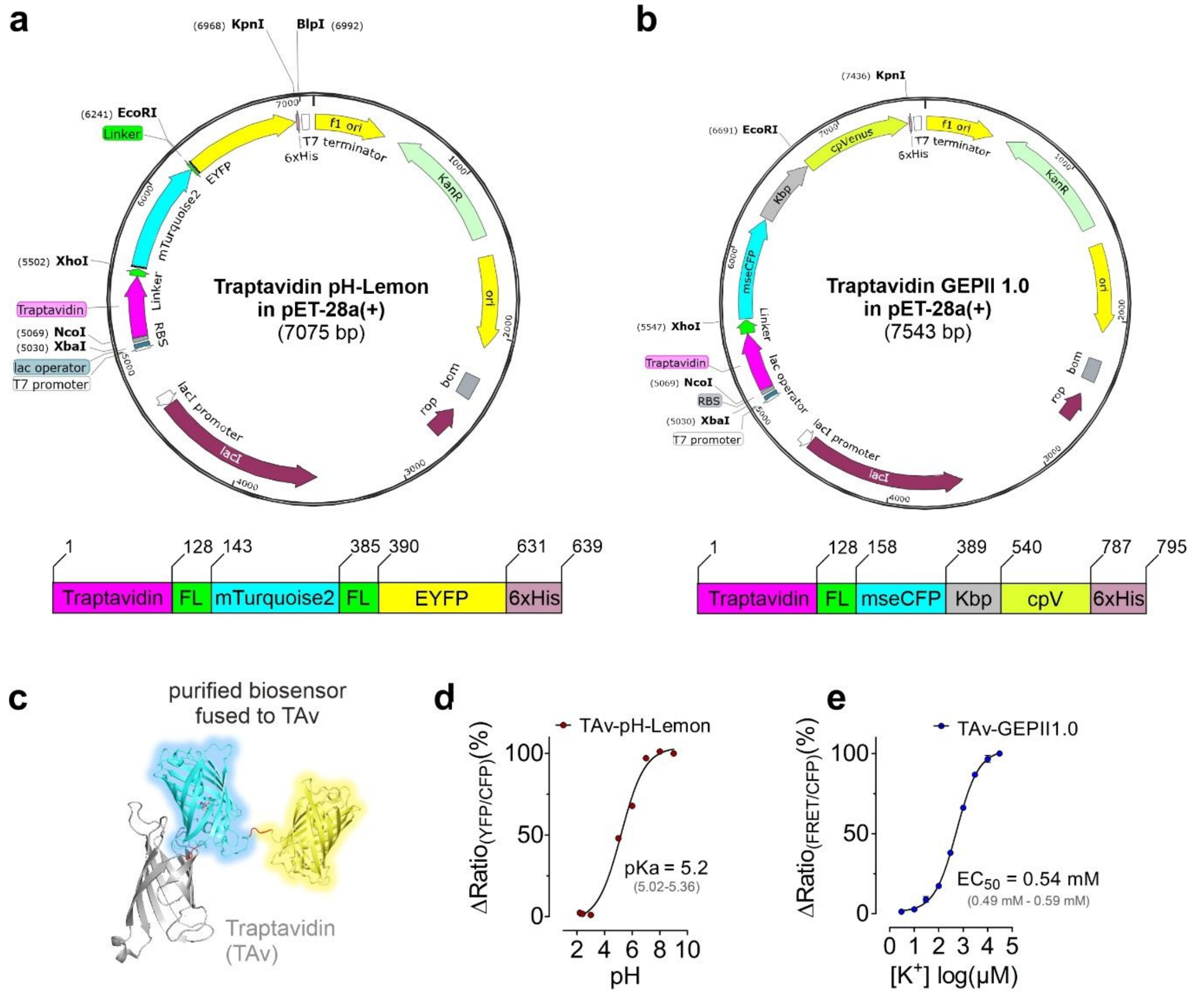

Figure S-3: Characterization of TAv-fused sensors in solution. (**a**) Schematic illustration of the pET28a(+) vector encoding for traptavidin (TAv)-pH-Lemon for expression in *E.coli*. pH-Lemon consists of mTurquoise2 (cyan) and EYFP (yellow) fused via a flexible linker (green). The construct is flanked by TAv (pink) fused to the N-terminal end of mTurquoise2 via a flexible linker (green) and by a 6xHIS tag (brown) fused to the C-terminal end of the EYFP. (**b**) Illustration of the pET28a(+) vector encoding for TAv-GEPII 1.0 for expression in *E.coli*. GEPII 1.0 consists of mseCFP (cyan) and cpV (yellow) fused to Kbp (grey). The construct is flanked by TAv (pink), fused to the N-terminal end of mseCFP via a flexible linker (green), and by a 6xHIS tag (brown) fused to the C-terminal end of the cpV. (**c**) Illustration of a sensor construct consisting of traptavidin (TAv, grey) on the N-terminal end followed by a FRET-based fluorescent biosensor (e.g. pH-Lemon or GEPII 1.0, cyan and yellow). The flexible linkers are shown in red. (**d**) Concentration-response curve of recombinant purified TAv-pH-Lemon *in vitro*. Data represent average ± SD of n=3 independent experiments. (**e**) Concentration-response curve of recombinant purified TAv-GEPII 1.0 *in vitro*. Data represent average ± SD of n=4 independent experiments.

Supplementary Figure 4

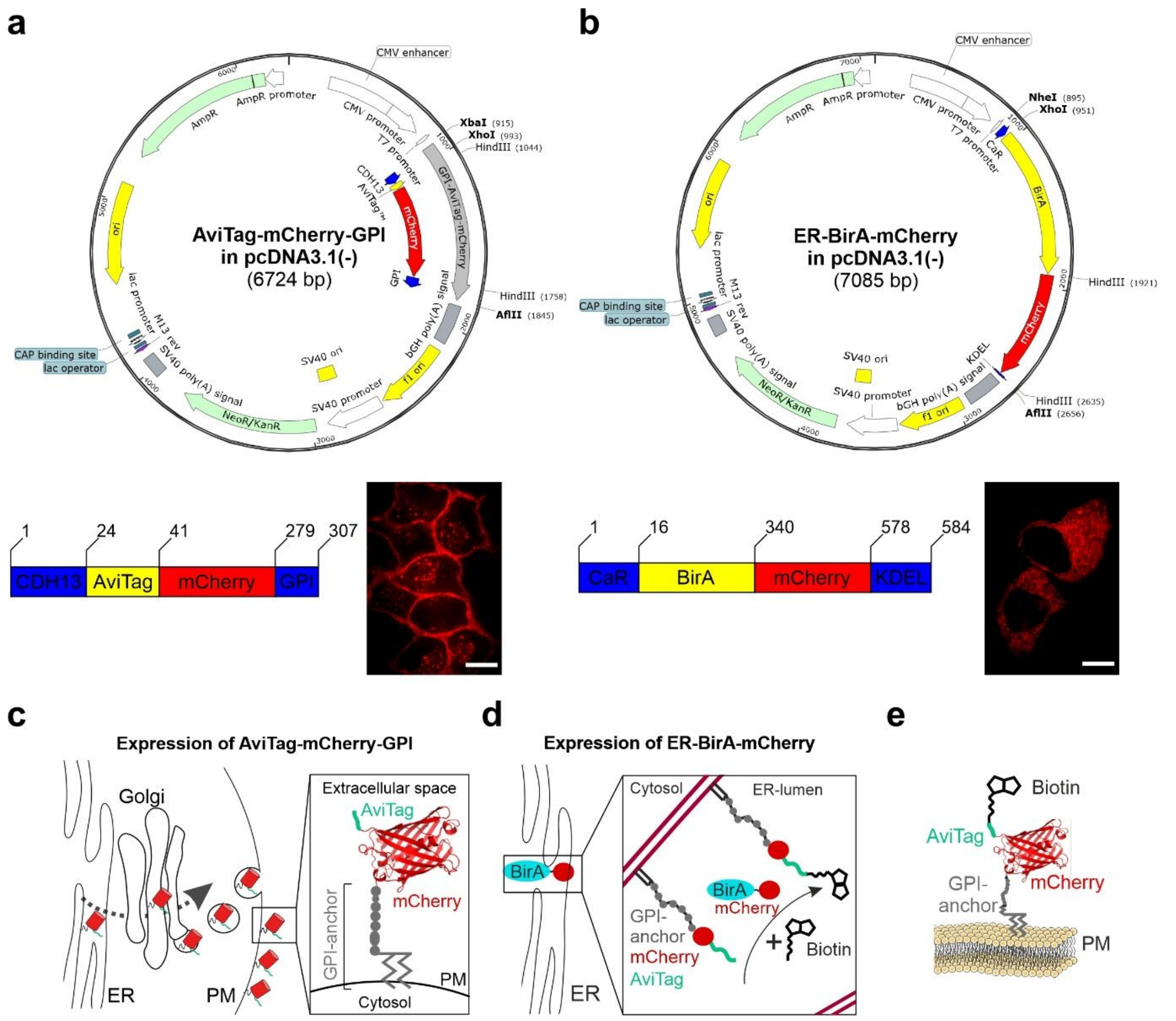

Figure S-4: Constructs and Scheme for the Immobilization of TAv-fused Biosensors. (**a**) Schematic illustration of the AviTag-mCherry-GPI construct in a pcDNA3.1(-) vector backbone. The N-terminal end of the AviTag (yellow) was fused to the leader signal of cadherin 13 (CDH13) (blue). The C-terminal end of the mCherry was fused to the GPI anchor attachment signal (GPI) (red) of CDH13. Representative ACLSM image of HeLa cells expressing the Avitag-mCherry-GPI construct. Scale bar represents 10 µm. (**b**) Schematic illustration of the ER-BirA-mCherry construct in a pcDNA3.1(-) vector backbone. The N- and C-terminal ends of BirA were fused to the first 16 amino acids of calreticulin as found in pDsRed2-ER vector and to the ER retention signal KDEL, respectively. Representative ACLSM image of HeLa cells expressing the ER-BirA-mCherry construct. Scale bar represents 10 µm. (**c**) Schematic representation of the intracellular trafficking of AviTag-mCherry-GPI. Upon membrane trafficking, the AviTag (of AviTag-mCherry-GPI) is biotinylated within the endoplasmic reticulum (ER) by the ER-located biotin ligase (ER-BirA-mCherry, panel **b**). (**d**) ER-targeted biotin ligase BirA biotinylates the AviTag (as a part of the AviTag-mCherry-GPI construct) while this construct travels through the ER upon membrane targeting. (**e**) The biotinylated AviTag is finally located at the cell membrane facing the extracellular space.

Supplementary Figure 5

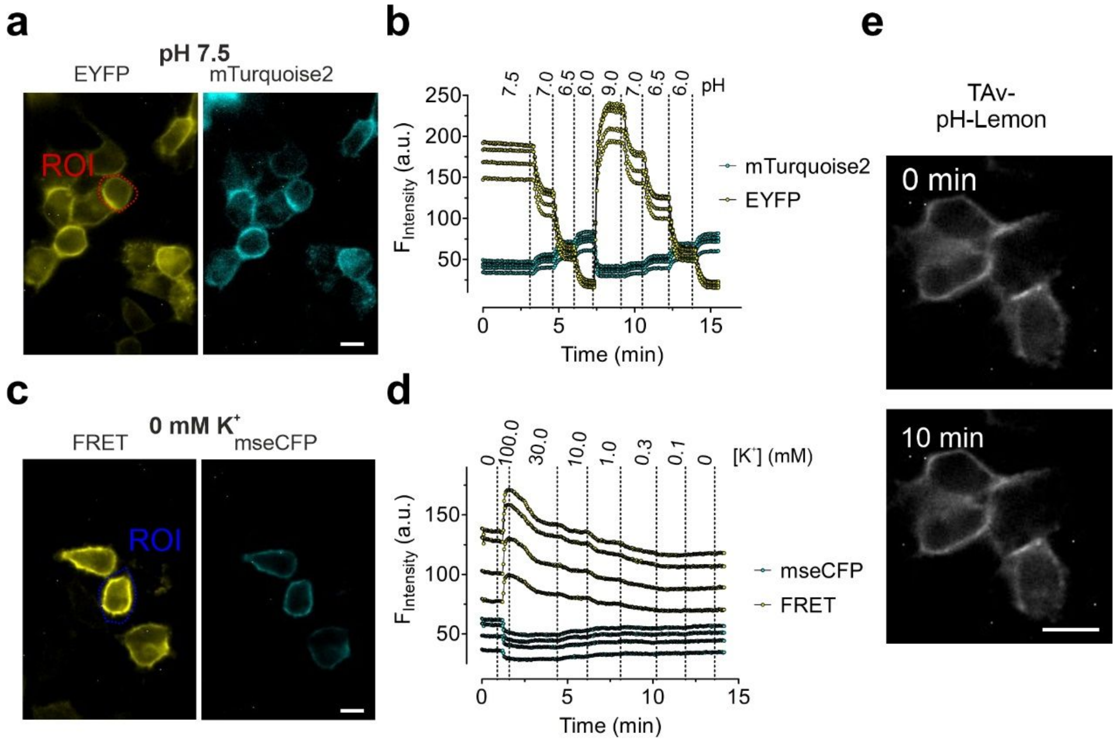

Figure S-5: Functionality of TAv-pH-Lemon and TAv-GEPII 1.0 after Immobilization. (**a**) Representative fluorescence wide-field images (EYFP and mTurquoise2) of HeLa cells incubated with TAv-pH-Lemon at an extracellular pH of 7.5. Scale bar represents 20 µm. (**b**) Panel displays representative single-cell fluorescence signals over-time of mTurquoise2 and EYFP of TAv-pH-Lemon immobilized on HeLa cells upon pH alterations as of regions (ROIs) as indicated in panel (**a**). n=3. (**c**) Representative fluorescence wide-field images (FRET and mseCFP) of HeLa cells incubated with TAv-GEPII 1.0 in the absence of $[K^+]_{ex}$. (**d**) Panel displays representative single-cell fluorescence signals over-time of mseCFP and FRET of TAv-GEPII 1.0 immobilized on HeLa cells upon altering $[K^+]_{ex}$. n=4. **(e)** Representative wide-field fluorescence images of HeLa cells with surface-immobilized TAv-pH-Lemon at 0 min and after 10 minutes of perfusion. Scale bar represents 20 µm.

Supplementary Figure 6

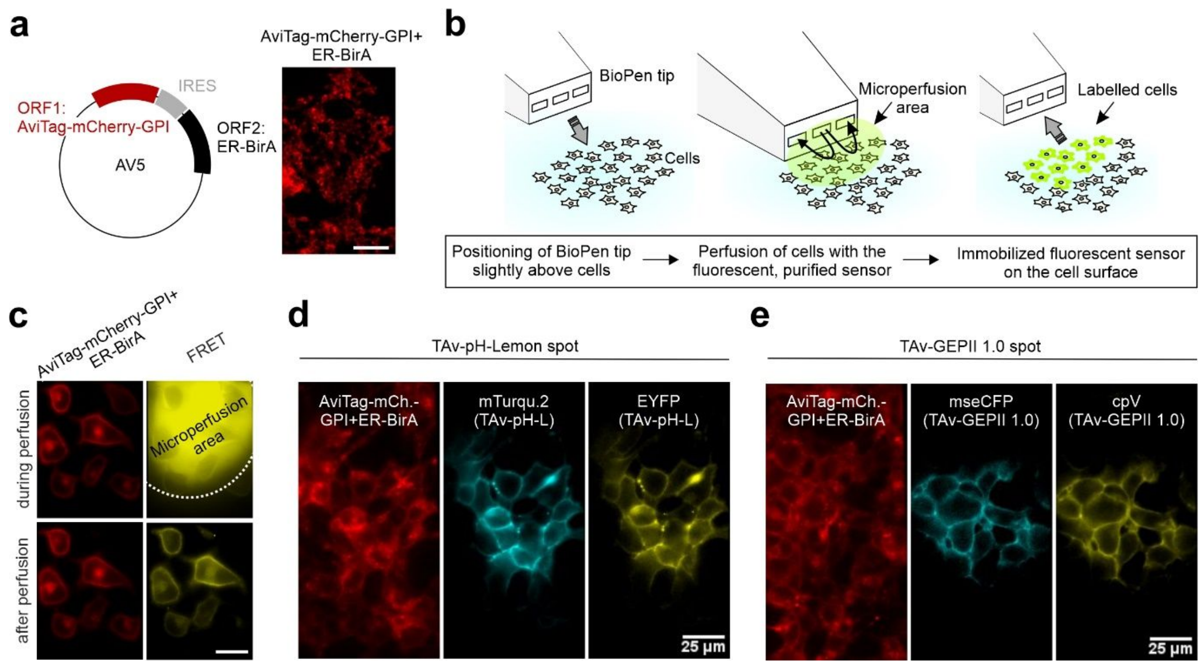

Figure S-6: Local immobilization of TAv-sensors using microperfusion. (**a**) Schematic illustration of an adenovirus type 5 (AV5) for infection of mammalian cells. The two open reading frames (ORF) for the AviTag-mCherry-GPI (ORF1, red) and ER-BirA (ORF2, black) are separated by an internal ribosomal entry site (IRES, grey) for co-expression. The image shows a representative widefield fluorescence image of INS-1 832/13 cells 2 days after infection. Scale bar represents 50 µm. (**b**) Illustration of sensor immobilization via microperfusion. The BioPen tip, which was loaded with the sensor, is placed adjacent to the cells attached to the culture dish. Fluid release (=sensor solution) and suction create a small perfusion area of adjustable size to specifically apply the sensor to cells of interest. After perfusion, the cells exposed to the sensor solution are loaded with the sensor. (**c**) Representative widefield mCherry and FRET images of HeLa cells infected with the virus during (upper images) and after (lower images) microperfusion. The perfusion area is indicated. Scale bar represents 20 µm. (**d**) Zoom-in at the cells microperfused with TAv-pH-Lemon (TAv-pH-L). Shown are fluorescence widefield images of INS-1 832/13 cells expressing Avitag-mCherry-GPI and ER-BirA (left image, AviTag-mCh.-GPI+ER-BirA), as well as the mTurquoise2 (middle image, mTurqu.2) and EYFP (right image) fluorescence of TAv-pH-Lemon. Scale bar indicates 25 µm. (**e**) Zoom-in at the cells microperfused with TAv-GEPII 1.0. Shown are fluorescence widefield images of INS-1 832/13 cells expressing Avitag-mCherry-GPI and ER-BirA (left image, AviTag-mCh.-GPI+ER-BirA), as well as the mseCFP (middle image) and cpV (right image) fluorescence of TAv-GEPII 1.0. Scale bar indicates 25 µm.

Supplementary Figure 7

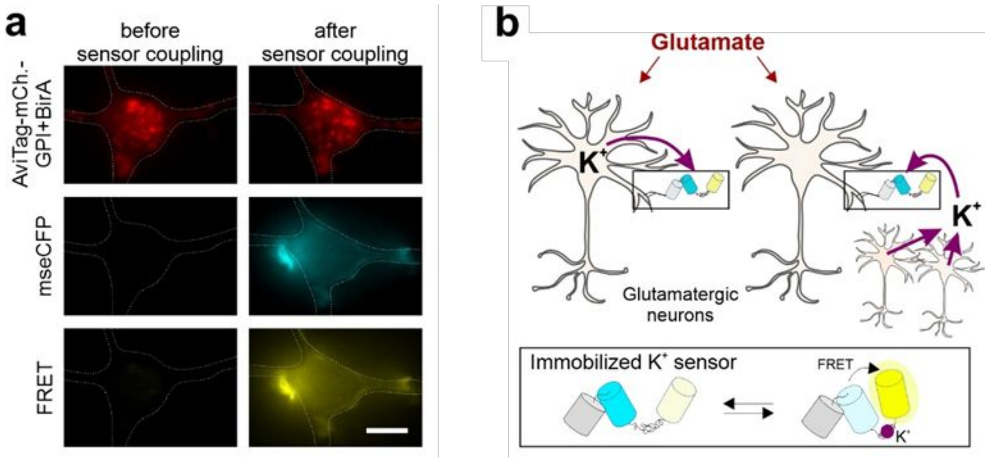

Figure S-7: Visualization of glutamate-induced K^+ efflux from primary neurons using surface-coupled TAv–GEPII 1.0. (**a**) Representative fluorescence wide-field images of a P0 rat cortical neuron transduced with an AV5 vector expressing AviTag-mCherry-GPI and ER-BirA either before (left images) or after the immobilization of TAv–GEPII 1.0 (right images). The scale bar represents 5 µm. (**b**) Schematic representation of glutamate-induced K^+ efflux from neurons upon glutamate stimulation locally measured by cell surface immobilized TAv–GEPII 1.0.

Supplementary Figure 8

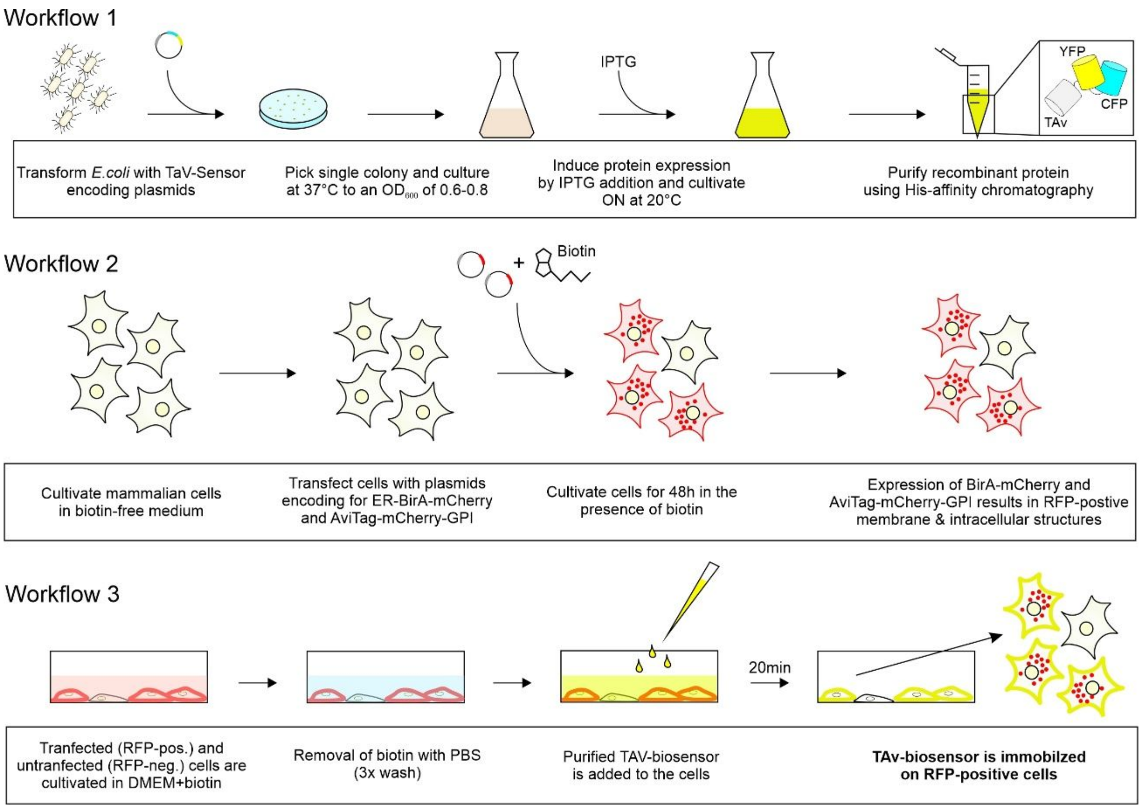

Figure S-8: Cell & sensor preparation and application to living cells.

WORKFLOW 1: Competent *E.coli* BL21 star cells were transformed with the sensor encoding plasmids for bacterial expression. The cells were spread onto LB agar plates containing kanamycin over-night. The next day, a single colony was picked transferred to LB medium with kanamycin. The culture was incubated at 37°C until an OD_{600} of 0.6-0.8 was reached. At this time point, IPTG was added to the culture to induce the expression of the sensor construct. From now on the culture was handled in the dark and the culture was incubated over-night at 20°C. The next day, the cells were lysed, and the protein was further purified using Ni-NTA affinity columns followed by a buffer exchange to obtain fluorescent biosensors in solution. **WORKFLOW 2**: To prepare the cells for immobilization, cells previously cultivated in a biotin-free medium were co-transfected with Avitag-mCherry-GPI and ER-BirA-mCherry and the medium was supplemented with biotin. After 48 hours of cultivation, RFP-positive cells were observed. **WORKFLOW 3**: These RFP positive cells were washed 3x with PBS to remove the residual biotin. The purified sensor (obtained from workflow 1) was added to the cells and the cells were incubated in the dark for 20 minutes. Subsequently, cells were washed with PBS or by perfusion to remove the unbound sensor. After washing, the cells were ready for measurements.

References

(1) Burgstaller, S. Visualization of local intracellular & cell surface cation alterations using fluorescent protein-based probes. Dissertation (available as full text after 04/2022 at https://online.medunigraz.at/mug_online/wbAbs.showThesis?pThesisNr=58221&pOrgNr=1#), Medical University of Graz, Graz, Austria, 2020.

(2) Gerndt, S.; Chen, C.-C.; Chao, Y.-K.; Yuan, Y.; Burgstaller, S.; Scotto Rosato, A.; Krogsaeter, E.; Urban, N.; Jacob, K.; Nguyen, O. N. P.*; et al.* Agonist-mediated switching of ion selectivity in TPC2 differentially promotes lysosomal function. *eLife* **2020**, *9*. DOI: 10.7554/eLife.54712.

(3) Bischof, H.; Rehberg, M.; Stryeck, S.; Artinger, K.; Eroglu, E.; Waldeck-Weiermair, M.; Gottschalk, B.; Rost, R.; Deak, A. T.; Niedrist, T.*; et al.* Novel genetically encoded fluorescent probes enable real-time detection of potassium in vitro and in vivo. *Nature communications* **2017**, *8*, 1422. DOI: 10.1038/s41467-017-01615-z

(4) Burgstaller, S.; Bischof, H.; Gensch, T.; Stryeck, S.; Gottschalk, B.; Ramadani-Muja, J.; Eroglu, E.; Rost, R.; Balfanz, S.; Baumann, A.*; et al.* pH-Lemon, a Fluorescent Protein-Based pH Reporter for Acidic Compartments. *ACS sensors* **2019**, *4*, 883–891. DOI: 10.1021/acssensors.8b01599
